# Decoding chemical interactions among pomegranate, *Aphis punicae*, and associated insects in Taif fields through open-loop stripping

**DOI:** 10.3389/fpls.2025.1541538

**Published:** 2025-06-26

**Authors:** Nour Houda M’sakni, Taghreed Alsufyani, Noura J. Alotaibi

**Affiliations:** ^1^ Department of Chemistry, College of Science, Taif University, Taif, Saudi Arabia; ^2^ Department of Biology, College of Science, Taif University, Taif, Saudi Arabia

**Keywords:** *Punica granatum L.*, *Aphis punicae*, non-targeted analysis, Taif governorate, multivariate statistical analysis, GC-MS headspace, open-loop stripping, integrated pest management

## Abstract

**Introduction:**

The escalating threat posed by *Aphis punicae* to *Punica granatum* cultivation underscores the urgent need for sustainable, ecologically sound alternatives to chemical pesticides. This study employs a non-targeted analysis of volatile organic compounds (VOCs) emitted by aphid-infested pomegranate (AIP), undergoing multitrophic interactions with natural enemies (*Coccinella undecimpunctata*) and mutualistic protectors (*Tapinoma magnum*). These VOCs are hypothesized to function as early biochemical markers of pest stress and semiochemical cues guiding insect behavior, offering potential integration into decision-support tools within integrated pest management (IPM) frameworks.

**Methods:**

VOCs were non-destructively collected using open-loop stripping and analyzed via gas chromatography–mass spectrometry under a metabolomics approach. Profiling was conducted across four ecological scenarios through integrated in-situ experimentation: (G1) AIP, (G2) AIP with ants, (G3) AIP with ants and ladybirds (24h), and (G4) AIP with ants and ladybirds (48h). Principal component analysis and heatmap clustering revealed scenario-specific VOC fingerprints.

**Results:**

In the two-trophic AIP system, early plant stress responses included suppressed emissions of β-farnesene and methyl salicylate, alongside elevated levels of caryophyllene, a compound often associated with herbivore activity. At 24h, under a tritrophic interaction, 4-heptanone, a key ant pheromone, was detected, suggesting a role in interspecies signaling or predator deterrence. After 48h, in the quadripartite trophic interaction, VOCs such as 1-ethyl-3-methylbenzene, 1,3,5-trimethylbenzene, and 1-methyl-1H-imidazole became dominant, likely reflecting aphid-induced signaling affecting multitrophic dynamics. In the same interaction, elevated levels of six herbivore-induced plant volatiles (6-HIPVs), methyl salicylate, β-caryophyllene, sabinene, limonene, pentadecane, and heptadecane, were observed, supporting indirect plant defense by attracting natural enemies. Bioassays showed that *C. undecimpunctata* exhibited significantly higher attraction to the mixture of 6-HIPVs compared to individual treatments with methyl salicylate or β-caryophyllene. The mixture elicited the highest behavioral response, indicating a synergistic effect among volatiles and supporting their role in enhancing predator attraction.

**Discussion:**

To transition from discovery to application, future research should focus on targeted analysis, compound-specific bioassays, optimized delivery systems, and open-field trials. Assessing these VOCs under varying agroecological conditions, along with evaluating economic feasibility, scalability, and regulatory pathways. This approach will be crucial for translating this chemical ecology framework into effective, climate-resilient IPM strategies tailored to the arid agroecosystems of the Taif and similar environments.

## 1 Introduction

Pomegranate (*Punica granatum*) is a culturally and economically significant fruit crop in Saudi Arabia, prized for its medicinal, nutritional, and pharmaceutical properties (Aboelhana et al., 2017). Its cultivation is primarily concentrated in the central-western agroclimatic zone, notably the Al Baha region and the Taif governorate of the Makkah Region, which together contribute over 35% of the country’s pomegranate production. With more than 200,000 trees yielding approximately 30,000 tons annually, this sector plays a vital role in local agricultural economies (Alghamdi et al., 2022; Hussain et al., 2024).

However, the productivity and sustainability of pomegranate cultivation are increasingly threatened by *A. punicae* Passerini (MZ091379), a specialist aphid species that causes substantial yield losses and compromises fruit quality (Alotaibi et al., 2023). By feeding on phloem sap, *A. punicae* weakens plant metabolism and facilitates the spread of plant pathogens (Farhan et al., 2024; Moawad and Al-Barty, 2011). Its honeydew secretions promote sooty mold formation, reducing photosynthetic efficiency (Hodgson et al., 2012). If left unmanaged, infestations can cause yield losses of up to 24 tons per hectare. In addition to this damage, *A. punicae* feeding alters plant biochemical signaling pathways, with cascading effects on multitrophic interactions and ecological stability (Lengai et al., 2020).Currently, chemical insecticides are the primary means of managing aphid infestations. However, their extensive use has raised concerns regarding pest resistance, non-target organism impacts, and environmental contamination (Ahmad et al., 2024). Studies have indicated that fruit crops in Saudi Arabia, such as oranges, grapes, and pomegranates, often exceed acceptable pesticide residue levels, with Hazard Index (HI) values surpassing the safety threshold of 1 (Alokail et al., 2024). These challenges underscore the urgent need for ecologically sound and sustainable pest management strategies tailored to local agroecosystems. Integrated pest management (IPM) provides a more balanced approach by combining cultural, biological, and chemical tools to maintain pest populations below damaging thresholds. Within this framework, the use of semiochemicals, particularly volatile organic compounds (VOCs), has emerged as a promising alternative for improving pest control specificity and reducing reliance on synthetic pesticides (Serdo, 2024). VOCs, including herbivore-induced (HIPVs) and oviposition-induced plant volatiles (OIPVs), play pivotal roles in mediating plant-insect and multitrophic interactions by deterring herbivores, disrupting mutualistic relationships, and attracting natural enemies (Conti et al., 2021; Cook et al., 2007; Zhou et al., 2024). Recent advances in semiochemical-based technologies, such as pheromone traps, targeted pesticide delivery, nanoemulsions, and controlled-release formulations are contributing to the development of more precise and environmentally sustainable pest management practices (Zhou et al., 2024).

In pomegranate systems, *A. punicae* not only causes direct plant damage but also interacts mutualistically with ants such as *Tapinoma magnum*, complicating biological control efforts (Campolo et al., 2015). Concurrently, the aphid shares its habitat with a diverse community of natural enemies, including syrphid flies, ladybirds (*Coccinella undecimpunctata*), and lacewings larvae (*Chrysoperla plorabunda*), which play critical roles in maintaining ecological balance and suppressing aphid populations (Elango and Sridharan, 2018; Michaud, 2001; Mohi-Ud-Din et al., 2019; Sayed et al., 2022). Understanding how plant-emitted VOCs influence these interactions is essential for developing biologically based IPM strategies (Taghreed Alsufyani et al., 2024).

This study is based on the hypothesis that pomegranate trees emit distinct VOCs in response to *A. punicae* infestation and its ecological interactions with predators and mutualistic ants. Using non-targeted chemical analysis, we characterize VOC emissions under varying ecological conditions to explore their ecological roles and practical applications. Specifically, we aim to: (1) identify key VOCs under these different conditions; and (2) evaluate their potential as tools for attracting natural predators, repelling pests, or enhancing monitoring systems within an Integrated Pest Management (IPM) framework. Our previous work on the bioactivity of endosymbiotic bacteria associated with aphids and their predators has revealed complex multitrophic interactions in the pomegranate system, further emphasizing the importance of chemical signaling in pest management (T. Alsufyani et al., 2023).

The findings from this study will support the development of environmentally friendly, VOC-based tools compatible with existing IPM strategies. Future phases will assess the practical applications of the identified compounds such as aphid alarm pheromones, predator-attractant volatiles, and pomegranate-induced defense signals, for use in pest trapping, herbivore deterrence, and behavioral modification. In addition, temporal variations in VOC emissions will be monitored to better understand their ecological dynamics. Ultimately, this research aims to evaluate the feasibility of integrating VOC-based strategies into Saudi Arabia’s agricultural and regulatory frameworks to support sustainable pomegranate production (Basu et al., 2021; Conboy et al., 2020; Vosteen et al., 2016).

## 2 Materials and methods

### 2.1 Study area

The study was conducted in the Taif governorate of Saudi Arabia, located at an elevation of approximately 1,879 meters (6,165 feet) on the slopes of the Sarawat Mountains (Alsherif and Fadl, 2016). The region experiences a semi-arid climate, with average annual temperatures around 22.2 °C (72.0 °F) and average annual rainfall of about 212 mm (8.3 inches). The unique topography and climate of Taif support diverse vegetation, including, sclerophyllous woodlands dominated by species such as *Juniperus procera*, *Olea europaea*, and various *Acacia* species (Sherif, 2015). This rich biodiversity contributes to complex ecological interactions between plants and insects, creating an environment conducive to pomegranate cultivation.

### 2.2 Pomegranate cultivar and agricultural practices

In this study, we utilized the ‘Taify’ cultivar of pomegranate (*Punica granatum L.*) (Ismail et al., 2017), sourced from Al-Sir farms (coordinates: N21.204753, E40.591998) in the Taif governorate. The ‘Taify’ cultivar is esteemed for its adaptability to local climate conditions and its significance in regional agriculture. This variety is characterized by its light-yellow peel with a sweet-sour taste and dark crimson arils, making it a valuable asset to the local economy and culture. Experiments were conducted on two-year-old pomegranate trees treated with fertilizers commonly used by local farmers to ensure healthy growth and fruit production. No additional fertilizers or chemical treatments were applied during the study period to ensure that the detected VOCs primarily originated from the aphid-plant interaction rather than external agricultural inputs.

### 2.3 Study organisms

This study focused on the chemical interactions among pomegranate (*Punica granatum*) plants, the aphid *Aphis punicae* (*A. punicae)*, its predator *Coccinella undecimpunctata* (*C. undecimpunctata*), and the ant *Tapinoma magnum* (*T. magnum*), which protects the aphids. ​*C. undecimpunctata*, commonly known as the eleven-spot ladybird, is a predominant natural enemy of aphids in the Taif governorate, with documented efficacy in preying upon *A. punicae*, highlighting its significant role in aphid population control. Similarly, *T. magnum* is frequently observed in pomegranate orchards, where it influences the dynamics between aphids and their natural enemies. Both species were collected from alfalfa and pomegranate farms within the Taif governorate, ensuring that our selection constitutes a random sample of all infected pomegranate trees in the area. This methodology enables us to extrapolate our findings to any infected pomegranate tree within the Taif governorate.

### 2.4 Collection and laboratory rearing protocols for *C. undecimpunctata* and *T. magnum*

Adult specimens of *C. undecimpunctata* and *T. magnum* were collected respectively from alfalfa and pomegranate farms in the Taif governorate (coordinates: N21.204753, E40.591998) during early morning hours to minimize stress. The individuals of these species were identified with accession numbers ON149797 and ON149799, respectively, according to our previous study (Alsufyani et al., 2023). The insects were placed into ventilated containers for transport to the laboratory. Upon arrival, adult *C. undecimpunctata* were sexed, and five pairs (one male and one female) were placed in a 15 × 15cm containers. Each container included a cotton pad soaked in a 1:1 honey-water solution and was supplemented with pollen grains to provide a suitable diet for the beetles. Daily inspections were conducted to monitor egg laying, any eggs found were transferred to Petri dishes lined with moist filter paper to support proper development. Once hatched, the larvae were transferred to new containers, and black cotton aphids were added as a food source. The rearing environment was maintained at 25 ± 2°C with a relative humidity (RH) of 50 ± 5% (Shalaby, 2021).

Individuals of *T. magnum* used in the experiment were fed sugar and kept in containers until used in the experiment. The ambient laboratory conditions were kept at approximately 28 ± 2°C with standard humidity levels.

All insect cultures were maintained under a 16:8 light-dark photoperiod, with regular maintenance procedures such as cleaning, food replenishment, and health monitoring to ensure the viability of the specimens for subsequent experiments.

### 2.5 Headspace analysis

#### 2.5.1 Experiment process

**Figure 1:**
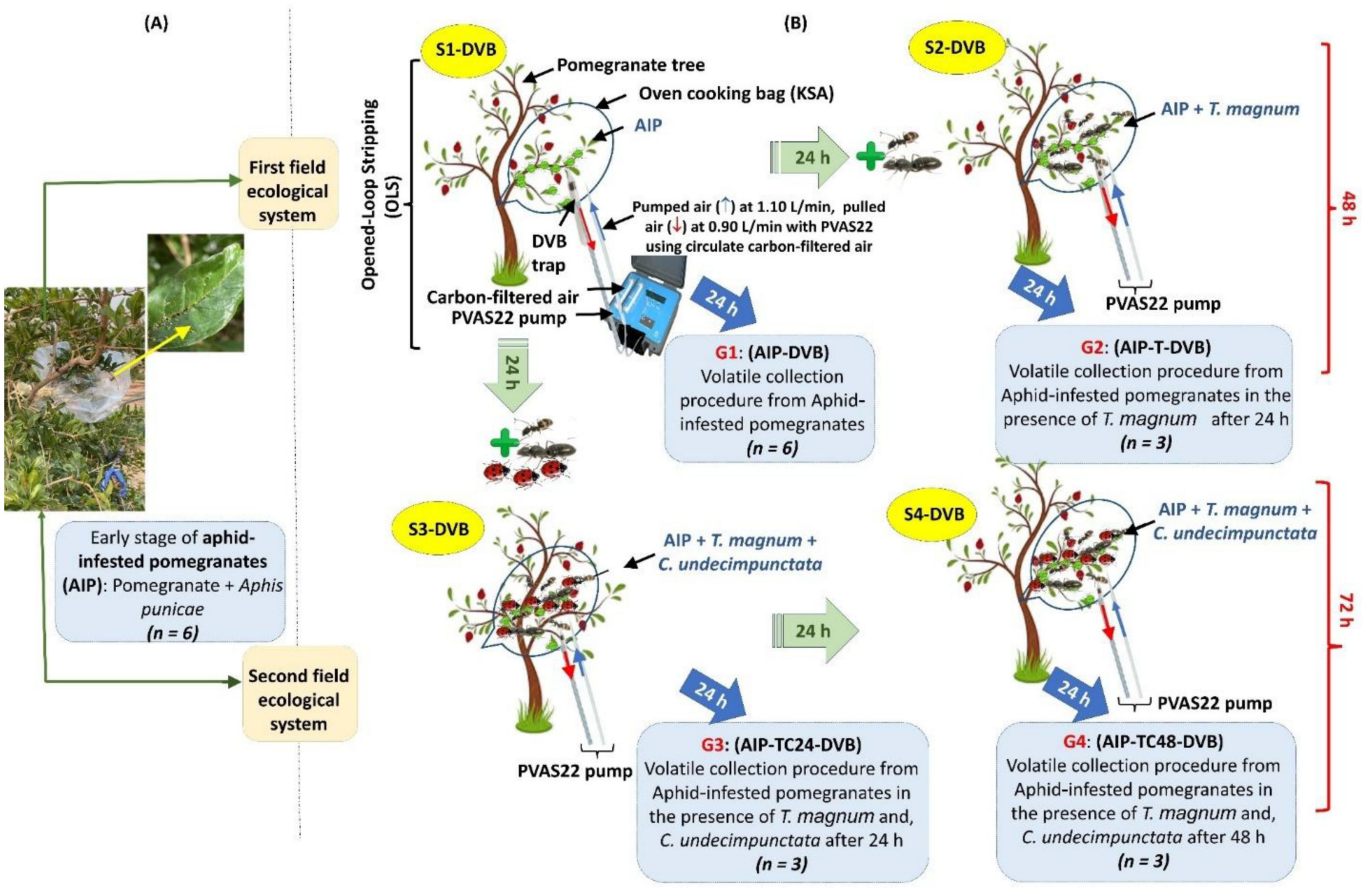
The experiment process involved collecting VOCs by OLS from two field ecological systems using four treatments (G1-G4). **(A)** plant material bagged and fastened to create an enclosed system for volatile collection; **(B)** the experiment process was started with AIP (G1: AIP), and ended with quadripartite communities (G3: AIP-TC24, G4: AIP-TC48), passing through G2: AIP-T, the AIP in the presence of *T. magnum* after 24 hours.

Airborne metabolites of pomegranate were characterized at different stages of aphid infection on a farm scale in May 2021 (**Figure 1**). The study focused on AIP at an early infection stage, particularly plants previously infected. Six branches (50 to 60 cm tall) from three pomegranate trees were selected from 3 trees, bagged, and fastened in natural oven cooking bags (King Saudi Arabia) to create an enclosed system that minimized the impact of abiotic and biotic factors (**Figure 1A**). VOCs were collected from various systems using DVB traps after 24 hours of interaction. To simulate natural conditions, two field ecosystems were created, representing a scenario involving infected plants, natural enemies, and protectors. The first field ecosystem consisted of the S1-DVB and S2-DVB stages (6 samples), while the second included the S1-DVB, S3-DVB, and S4-DVB stages (9 samples). The stages are as follows: (a) the S1-DVB stage, where aphids infest pomegranates (AIP); (b) the S2-DVB stage, where 20 *T. magnum* are added to three S1-DVB stages after 24 hours; (c) the S3-DVB stage, where 20 *T. magnum* and 10 female *C. undecimpunctata* are added simultaneously to three other S1-DVB stages after 24 hours; and (d) the S4-DVB stage, which mirrors the quadripartite community setup of S3-DVB after 48 hours (**Figure 1B**). Each stage had three independent biological replicates, with six replicates initiated for the S1-DVB stage.

#### 2.5.2 VOCs sampling

The collection, separation, identification, and quantification of VOCs are crucial steps in VOC analysis. One of the most commonly used methods for VOC collection, especially for microorganisms, is closed-loop stripping (CLS) analysis, as outlined by Grob (Badra et al., 2021; Grob et al., 1975; Kunert et al., 2009), (also known as static headspace), and open-loop stripping (OLS) analysis, following the method by Hans (Borén et al., 1982; Tholl et al., 2006). (also known as dynamic headspace). In this study, VOCs were collected from various stages of the AIP using the OLS headspace sampling method, as described by Effah et al. (2020). Pomegranate branches that showed no visible signs of damage from insects or pathogens were selected and placed in oven-safe cooking bags (KSA). Both sample and blank experiments (empty bags) were analyzed using GC-MS and OLS.

Each bag was fitted with an inlet tube and an outlet tube secured with cable ties. A portable Volatile Assay System (PVAS22 pump, Rensselaer NY) was employed to circulate carbon-filtered air through the bag. The incoming air was pumped at a rate of 1.10 L/min, while the outgoing air was pulled at 0.90 L/min, creating slight overpressure to prevent contamination from entering the bag. VOCs emitted from the field ecological systems within the AIP were trapped in divinylbenzene (DVB) polymer traps (VAS HayeSep Q Volatile Trap, Tip diameter/Length: 4 mm/8.8 cm, spherical particles of divinylbenzene polymer: 30 mg +/- 0.3 mg, Adsorbent surface area per trap: 17.5 m²; VAS, Rensselaer NY) attached to the outlet (pull) tubes.

At the beginning of the process, DVB traps were connected to the PVAS22 pump, which circulated clean air into the bag’s headspace while simultaneously pulling the air through the pre-cleaned DVB traps. The traps were pre-cleaned following established protocols (Light et al., 2020), which involved flushing them sequentially with methanol, acetone, and HPLC-grade hexane. Compressed purified inert gas (such as nitrogen) was then used to push the solvent through the trap at 10 to 20 psi, drying the adsorbent. The DVB traps were connected to the pump via airtight polypropylene tubing (**Figure 1B**). The system was run for 24 hours, after which the DVB traps were carefully removed, wrapped individually in labeled aluminum foil, and stored in a cooler for transport to the laboratory. This one-hour transport period was necessary to prevent contamination and evaporation of the collected volatiles.

#### 2.5.3 Sampling vs. treatments and ecological systems of AIP

The DVB polymer VOC traps were eluted with 60 µL of ethyl acetate (divided into three 20 µL aliquots). The samples were then analyzed using GC-MS or stored at −80°C for later analysis. VOCs collections were categorized as follows: G1: AIP for plants infected with aphids, and G2: AIP-T for aphid-infected plants in the presence of *T. magnum* after 24 hours. In later stages, samples were labeled G3: AIP-TC24 and G4: AIP-TC48 for plants exposed to both *T. magnum* and *C. undecimpunctata* after 24 hours and 48 hours, respectively (**Figure 1**).

In this study, VOC sampling was performed at 24-, 48-, and 72-hours post-infestation using an open-loop stripping (OLS) system, a method consistent with established protocols in plant-insect interaction research. For instance, a study on *Arabidopsis* exposed to aphid feeding used headspace solid-phase microextraction (SPME) with gas chromatography–mass spectrometry (GC-MS) to analyze VOC emissions over three-time intervals: 0–24, 24–48, and 48–72 hours (Truong et al., 2014). Similarly, research on apple plants infested with *Pandemis heparana* larvae employed closed-loop stripping analysis (CLSA) to collect VOCs at 1, 24, and 48-hour intervals (Giacomuzzi et al., 2016). These studies validate our selected sampling time points and the use of OLS for VOC collection in insect-infested plants.

#### 2.5.4 Gas chromatography-mass spectrometry analysis conditions

Samples were analyzed using a Clarus 580 Gas Chromatograph (GC) coupled with a Clarus 560S Mass Spectrometer (MS) (Agilent Technologies, Santa Clara, USA). The GC was equipped with a non-polar Elite-5ms Capillary Column (30 m × 0.25 mm ID, 0.25 µm film thickness, Agilent Technologies). Helium served as the carrier gas at a constant flow rate of 1 mL/min. A 1 µL aliquot of each sample was injected in split mode with a 1:5 ratio, using an injector temperature of 220°C and a pre-dwell time of 0.1 minutes. The oven temperature program commenced at 40°C for 3 minutes, increased at 2.8°C/min to 130°C, then at 2.4°C/min to 180°C, and finally at 6°C/min to 250°C, with a total GC runtime of 67 minutes. The strategy of identifying compounds based on reverse matching for untargeted analysis was adopted by Alotaibi et al. (2023).

Mass spectrometry (MS) was performed with an electron energy of 70 eV, a trap current emission of 100 µA, a source temperature of 150°C, and a transfer line temperature of 280°C. The MS detector operated in scan mode over a mass-to-charge ratio (m/z) range of 40–450 amu. Data acquisition and analysis were conducted using Turbomass software (PerkinElmer), and compound identification was achieved by comparing mass spectra to the NIST 14 library (Gaithersburg, MD, USA). Identification was further verified by co-injecting authentic standards alongside the samples Alotaibi et al. (2023).

#### 2.5.5 GC–MS data processing for multivariate analysis

Chromatograms were manually inspected, and the relative abundance (%) of each peak was calculated by dividing the area of each individual peak by the total sum of all detected peak areas, then multiplying by 100. The mean of the replicates was used for subsequent statistical analysis. Prior to statistical analysis, MS data underwent several preprocessing steps, as follows: 1) inclusion of negative controls, 2) peak picking/detection, 3) automated peak matching, and 4) data matrix generation (Sun and Xia, 2024). These steps are described below: 1) **Negative controls:** Air samples from empty bags were collected in triplicate to exclude potential contaminants. Chromatogram samples were cleaned by subtracting the areas corresponding to contaminants signals. 2) **Peak picking/detection:** Each ion in the sample was identified and assigned to a feature (m/z-RT pair), excluding any peaks below an arbitrarily defined area threshold. This involved aligning peaks, detecting, and performing quality control (data cleanup) by removing non-representative peaks. Typically, peaks with m/z values below 300 and scan numbers less than 7 were excluded, while peaks with local maxima were retained. 3) **Automated peak matching:** Peaks were matched based on their spectral signatures, merging them as necessary to ensure accurate identification. 4) **Data matrix construction:** The integrated peak height or area for each feature was assigned to a data matrix for further processing. This matrix included annotated biomarkers and their relative abundance percentages across samples, with each row representing a sample and each column representing a specific stage of the field ecological systems (**Table 1**).

**Table 1:** A data matrix generated of each stage of field ecological systems using headspace OLS and GC-MS analysis.

(n = 15) x (m = 30)	Bio 1	Bio 2	…….	Bio 30
S1-DVB_1_	% relative abundance …….			
S1-DVB_2_				
…….				
S4-DVB_14_				
S4-DVB_15_				

### 2.6 Statistical analysis

**Figure 2:**
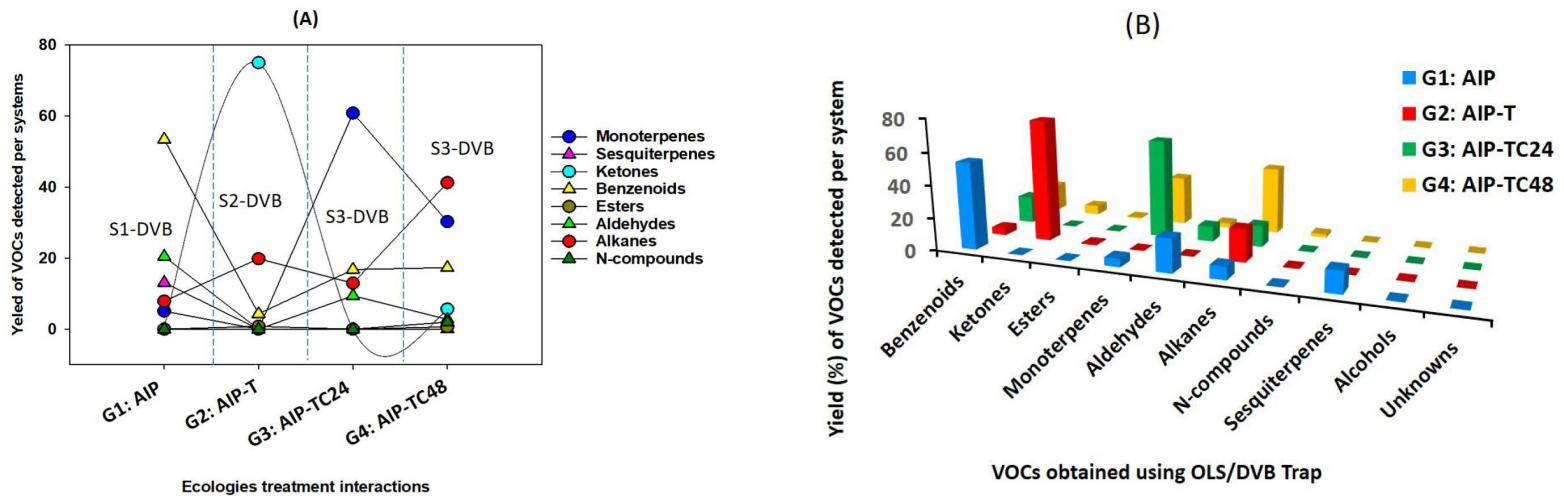
**(A)** Distribution of VOC families among all study systems (S1-DVB, S2-DVB, S3-DVB, and S4-DVB) in terms of ecological treatment interactions, **(B)** A histogram displays the chemical classification of quadruplet interactions VOCs in various experimental settings.

For non-target analysis, VOC profiling across all ecological systems included 30 VOCs, selected based on their consistent presence in samples from at least one species. One replicate of *Fragilis* that did not produce the chosen compounds was excluded from the analysis. The VOC data were converted into relative abundance percentages to ensure normality. The percentage of each major VOC class (**Table 2**) was calculated by summing the specific VOC pairs for each group and then dividing by the total blend (**Table 2**; **Figures 2A, B**). Statistical analyses were performed using PCA and the Analytical Hierarchy Process (AHP) through SRplot, an online data visualization platform (version 2023, PMID: 37943830, University of Nebraska-Lincoln, UNITED STATES) (Tang et al., 2023). Before conducting PCA, data preprocessing included calculating the average values for each sample group and excluding compounds with zero values. Graphical representation of the results was created by importing the first two principal axes, F1 and F2, along with sample coordinates, into SigmaPlot (version 11.0, Systat Software, USA). Biomarkers were screened for significant correlation coefficients using PCA axes, with compounds showing a correlation coefficient (|r| ≥ 0.1) retained as significantly characteristic. Both PCA and AHP were used to illustrate the variability of VOCs across different insect species and host plants.

**Table 2:** VOCs (n=30) were released into the headspace at two field ecological systems of aphid-infested pomegranate (AIP) in May 2021, followed by the first ecological system with the addition of *T. magnum* after 24 hours to AIP (6 samples).

VOC emissions	Biomarkers	RT (min)	Stage of field ecological systems	S1-DVB	S2-DVB	S3-DVB	S4-DVB
			Chemicals/samples	G1: AIP (*n* = 6)	G2: AIP-T (*n* = 3)	G3: AIP-TC24 (*n* = 3)	G4: AIP-TC48 (*n* = 3)
Exclusive AIP	Bio 5	26.94	Bio5: 1-Methyl-3-tert-butylbenzene	15.00	0	0	0
	Bio 6	26.95	Bio6: m-Ethylcumene	10.18	0	0	0
	Bio 11	10.6	Bio11: 3-Carene*	2.95	0	0	0
	Bio 15	30.9	Bio15: (+)-4-Carene	2.12	0	0	0
	Bio 17	16.35	Bio17: alpha-Methylbenzeneacetaldehyde	0.06	0	0	0
	Bio 18	13.19	Bio18: 2,2,4-trimethyl-Pentane	3.54	0	0	0
	Bio 22	37.47	Bio22: Unknown alkane-3	0.02	0	0	0
	Bio 23	37.71	Bio23: Unknown alkane-4	0.02	0	0	0
	Bio 27	33.99	Bio27: Caryophyllene*	13.07	0	0	0
	Bio 28	35.39	Bio28: β -Farnesene*	0.05	0	0	0
AIP+Lb	Bio 1	8.04	Bio1: o-Xylene	3.71	0.00	3.93	4.63
	Bio 16	12.09	bio16: Benzaldehyde*	20.41	0.00	9.41	2.77
	Bio 7	27.91	Bio7: 2-Methylnaphtalene	14.92	0.00	0.00	2.17
	Bio 21	28.79	Bio21: Tridecane	4.32	0.00	0.00	8.90
Common	Bio 2	8.91	Bio2: p-Xylene	9.63	4.32	8.73	5.71
Exclusive AIP + An	Bio 9	9.97	bio9: Methyl methanoate	0.00	0.80	0.00	0.00
	Bio 19	18.9	Bio19: Unknown alkane-1	0.00	19.84	0.00	0.00
Exclusive An +Lb	Bio 8	8.24	Bio8: 4-Heptanone	0.00	75.04	0.00	5.66
Exclusive Ladybird/Aphid interaction	Bio 3	11.95	Bio3:1-Ethyl-3-methylbenzene	0.00	0.00	1.02	1.29
	Bio 4	13.49	Bio4:1,3,5-Trimethylbenzene	0.00	0.00	3.12	3.53
	Bio 10	23.64	Bio10: Methyl Salicylate*	0.00	0.00	0.00	0.68
	Bio 12	12.55	Bio12: Sabinene	0.00	0.00	0.00	2.64
	Bio 13	15.2	Bio13: Tricyclene	0.00	0.00	60.84	0.00
	Bio 14	15.23	Bio14: Limonene*	0.00	0.00	0.00	27.62
	Bio 20	19.22	Bio20: Unknown alkane-2	0.00	0.00	12.95	26.86
	Bio 24	37.83	Bio24: Pentadecane*	0.00	0.00	0.00	2.78
	Bio 25	46.53	Bio25: Heptadecane*	0.00	0.00	0.00	2.75
	Bio 26	14.26	Bio26: 1-Methyl-1H-imidazole	0.00	0.00	0.00	2.00
	Bio 29	36.28	Bio29: β-helmiscapene (β-Selinene)	0.00	0.00	0.00	0.02
	Bio 30	48.07	bio30: Unknown-4	0.00	0.00	0.00	0.01
Total monoterpenes				5.07	0.00	60.84	30.26
Total sesquiterpenes				13.13	0.00	0.00	0.02
Total ketones				0.00	75.04	0.00	5.66
Total aldehydes				20.47	0.00	9.41	2.77
Total esters				0.00	0.80	0.00	0.68
Total benzenoids				53.44	4.32	16.80	17.32
Total N-compounds				0.00	0.00	0.00	2.00
Total alkanes				7.90	19.84	12.95	41.28
Total unknown compounds				0.00	0.00	0.00	0.01

The second ecological system can be achieved by adding *T. magnum* and *C. undecimpunctata* at the same time to AIP (9 samples). Metabolites were extracted by OLS, and identified respectively by GC-MS and NIST library.

AIP, Aphid-infested pomegranate; Lb, ladybird; An, ant, common: AIP + An + Lb, G1: AIP (S1-DVB): Aphid-infested pomegranate after 24h, G2: AIP-T (S2-DVB): Aphid-infested pomegranate (after 48h) + Ant (after 24h), G3: AIP-TC24 (S3-DVB): Aphid-infested pomegranate + Ant + Ladybirds (after 24h), G4: AIP-TC48 (S4-DVB): Aphid-infested pomegranate + Ant + Ladybirds (after 48h) were collected by OLS systems-DVB. RT: retention time; * Several key compounds were verified by comparison with authentic standard (Alotaibi et al., 2023). All metabolites marked with a had reverse matches between 700 and 990.

#### 2.6.1 Principal component analysis strategy

**Figure 3:**
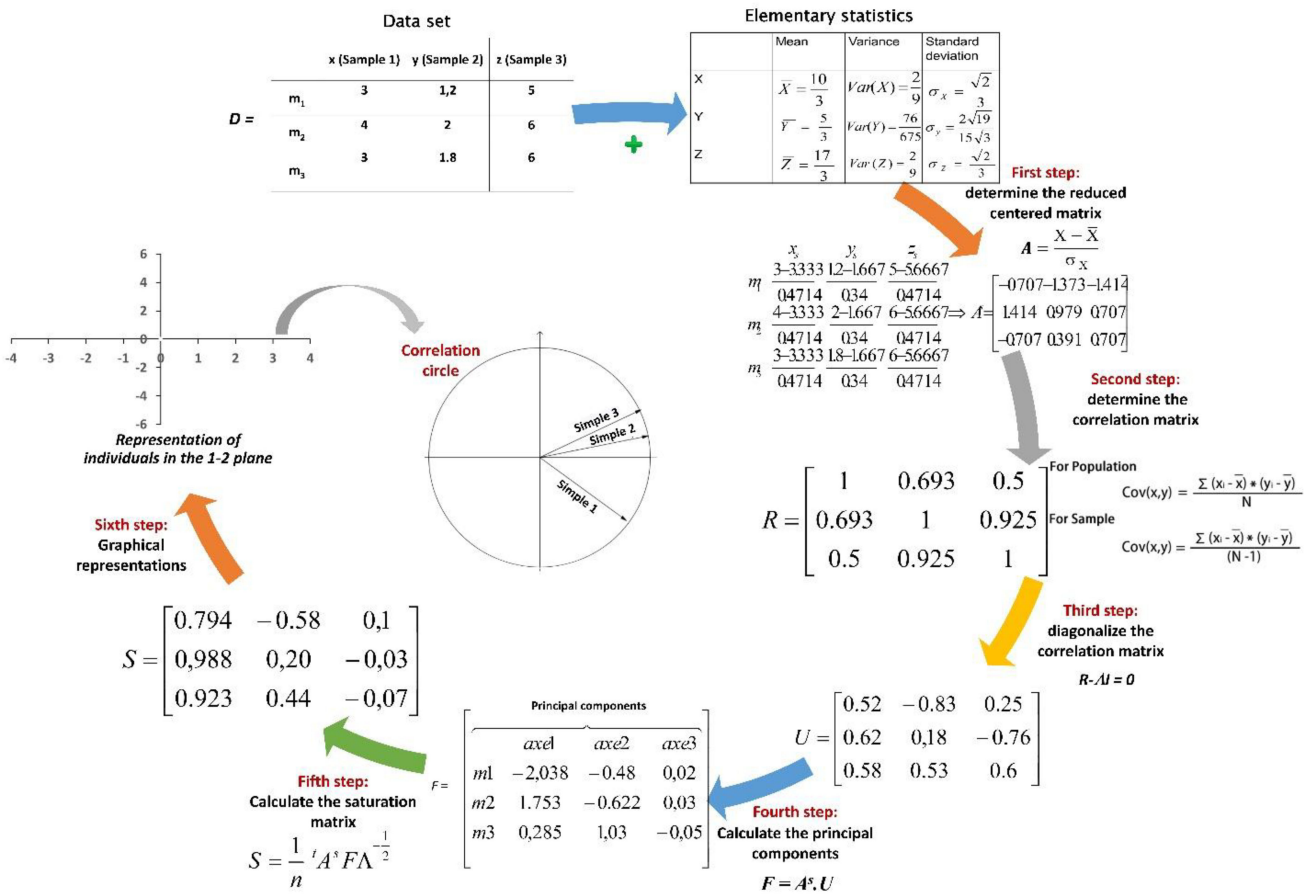
The steps and equations of PCA; x, y, and z, samples in a data set; m1, m2, and m3, populations in data set; n, number of value in the data set (n=3 in this example); D, data set; A: standardized data set; R, covariance matrix for the whole data set; U, eigenvectors; Λ, corresponding eigenvalue associated with eigenvector ν of R; S: data transformation F: principal components; S, saturation matrix.

PCA was introduced in 1901 by Pearson (1901). This technique is a powerful tool for analyzing quantitative data, whether continuous or discrete. PCA simplifies the description of a set of interrelated quantitative variables (Xp) by transforming the original variables into a new set of uncorrelated variables called principal components. Each principal component is a linear combination of the original variables (Hamdi et al., 2014). The primary goal of PCA is to identify the most efficient low-dimensional representation of the variance within a multivariate dataset. As one of the classic methods for dimensionality reduction, PCA reduces the number of variables or dimensions without losing significant information. The PCA process involves several phases: first, calculating statistical variables, followed by constructing a correlation matrix among the variables, and finally determining the eigenvalues. A detailed description of these steps, including the associated equations, can be found below (**Figure 3**).

#### 2.6.2 Analytical hierarchy process strategy

Saaty (1980) introduced the Analytical Hierarchy Process (AHP) as a method for addressing complex problems within a hierarchical or network structure. This comprehensive aggregation method is used to assess the relative importance of various criteria in relation to a specified objective. AHP is based on a systematic approach that analyzes complex decision-making problems by breaking them down into manageable, understandable steps. Each step is carefully evaluated, and the results are combined to form a logical solution. Using this approach, criteria can be selected and compared through pairwise evaluation of pre-established alternatives. The detailed description of the method, including the steps and equations, was provided by Velmurugan et al. (2011).

### 2.7 Bioassay protocol: Petri dish behavioral testing *C. undecimpunctata* attraction to plant volatiles

**Figure 4:**
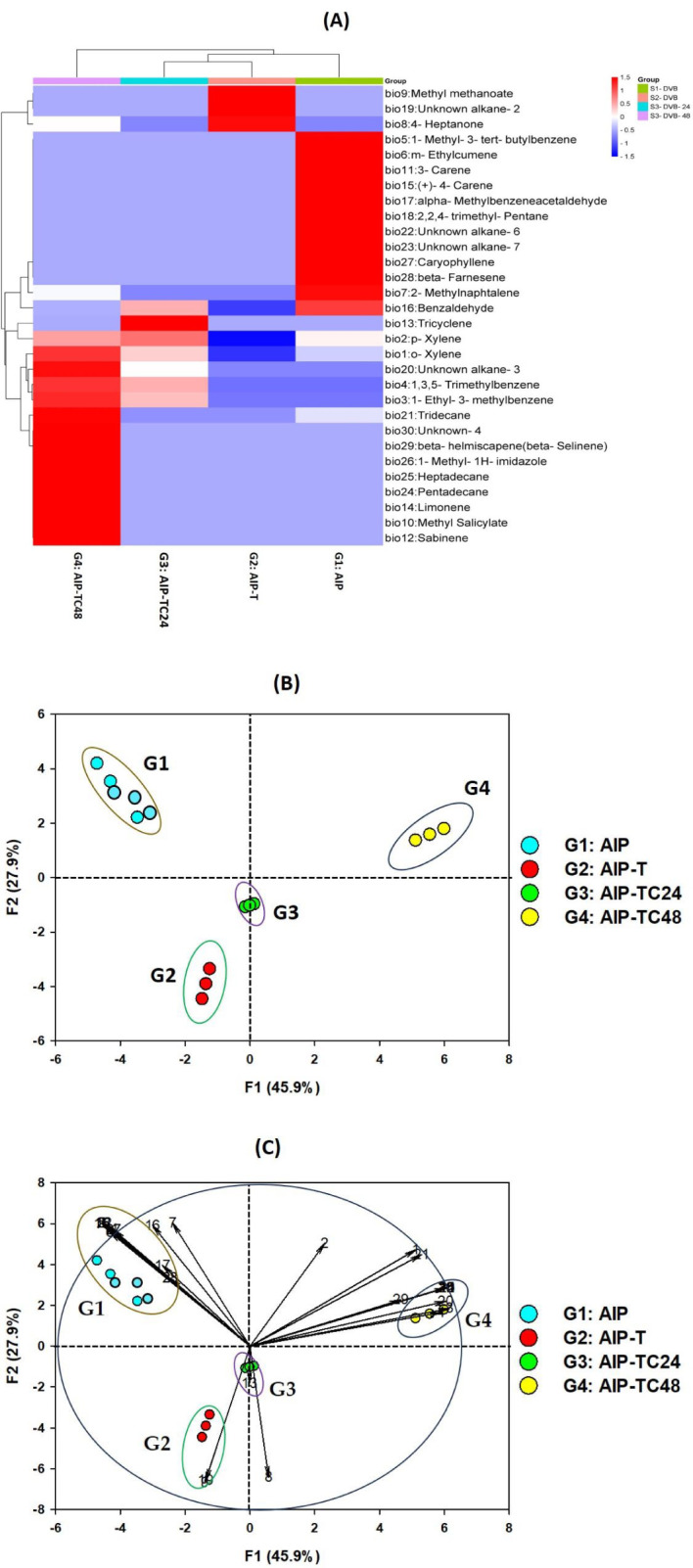
PCA and heatmap based on the relative percentages of VOCs from the different ecologies system of the quadruplet interaction plant-aphid-ant-ladybird: **(A)** Heatmap representing the hierarchical clustering of volatiles with significantly modified abundance between the dierent ecological systems. Each line represents a mass feature (volatile compound) and each column represents a sample. The color indicates the normalized intensity and the dendrograms display the similarity based on the Pearson algorithm. The identity of the VOCs is listed in **Table 2**; **(B)** correlations circle of the VOCs variables on the two principal components F1 and F2; **(C)** projection of the 12 samplings of VOCs from the four ecological systems using two DVB fibres on the two axes F1 and F2; PCA score plots, highlighting cluster of volatiles attributable to different infestation statuses (ellipses = 73.8% of confidence) and PCA loading plot, showing variable correlations with the first and second principal component were together bi plotted with SRplot.

A standard 9 cm Petri dish was divided into three functional areas: a compound area (treatment zone), a control area, and a neutral central area. In the compound area, 10 µL of a volatile solution (methyl salicylate, caryophyllene, or a mixture of six compounds mixture (6-HIPVs): methyl salicylate, caryophyllene, sabinene, limonene, pentadecane, and heptadecane), each diluted to 1% (v/v) in hexane, was applied to filter paper. In the control area, 10 µL of pure hexane was applied to filter paper (**Figure 4**). The following chemical compounds were obtained from certified suppliers: n-hexane (CAS: 110-54-3; purity ≥ 96%), methyl salicylate (CAS: 119-36-8; purity ≥ 99%), β-caryophyllene (CAS: 87-44-5; purity ≥ 98%, GC), sabinene (CAS: 3387-41-5; purity ≥ 98.5%) from CPAChem (EU), limonene (CAS: 5989-27-5; purity 97%), pentadecane (CAS: 629-62-9; purity ≥ 99%), and heptadecane (CAS: 629-78-7; purity ≥ 99%) from Sigma-Aldrich (St. Louis, MO, USA). All compounds were used without further purification.

After 10 minutes of solvent evaporation, 10 aphids (*A. punicae*) were placed in both the treatment and control areas. One adult *C. undecimpunctata*, starved for 12 hours prior to the assay, was then released at the center of the Petri dish, equidistant from both zones (**Figure 4**).

The assay was conducted under controlled environmental conditions (25 ± 2 °C, 50 ± 5% RH, 2000 lux), and the ladybird’s behavior was monitored for 30 minutes. The time spent in each zone and the number of aphids consumed were recorded (Siddique et al., 2019).

**Figure 5:**
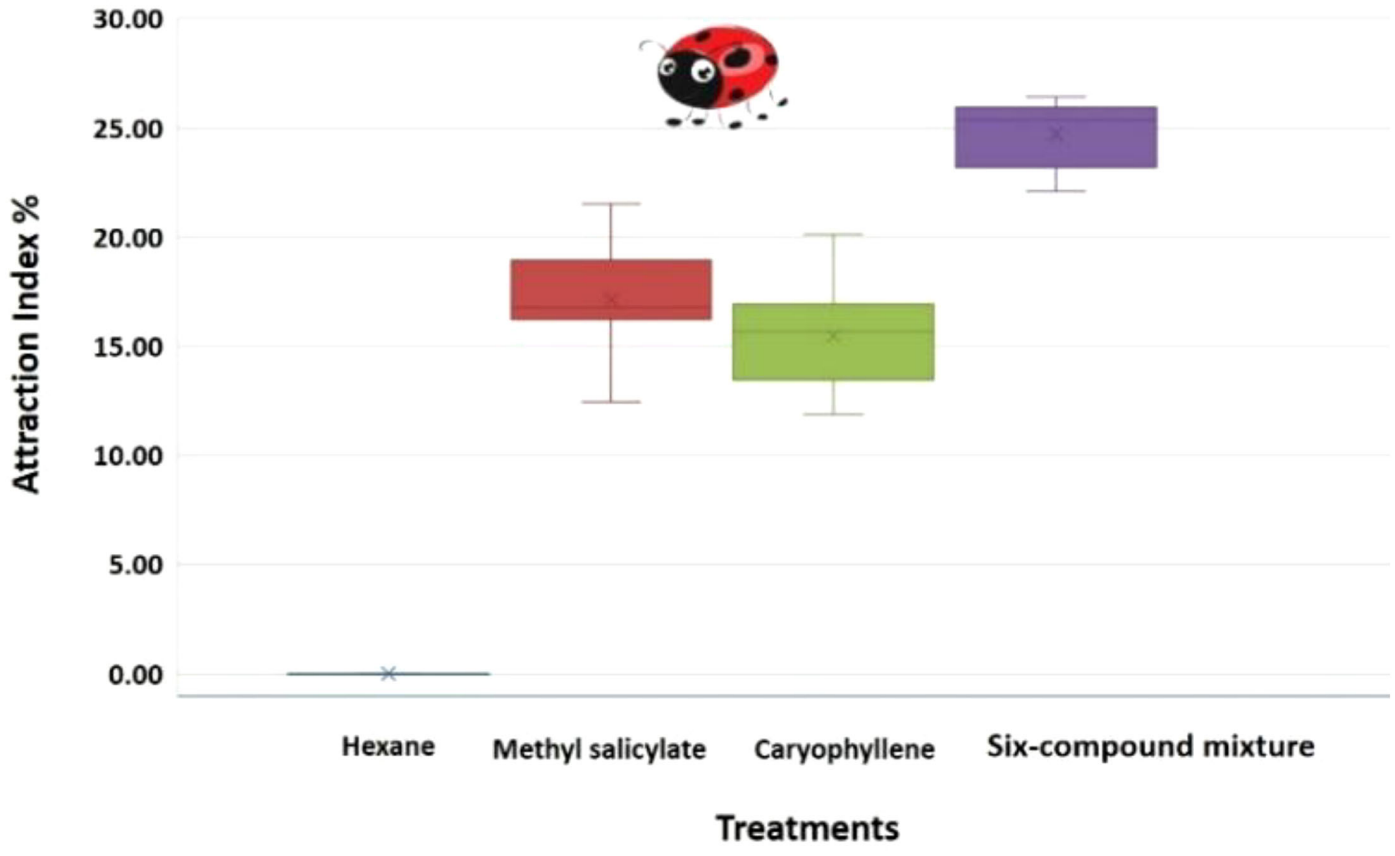
Boxplot of the attraction index (%) of *Coccinella undecimpunctata* in response to three volatile treatments: methyl salicylate, caryophyllene, six-compound mixture (6-HIPVs) and a control (n-hexane) (N = 10; 20 insects per test). Calculated using Equation 1; Wilcoxon test, *P*< 0.05; α = 0.05.


**Figure 6:**
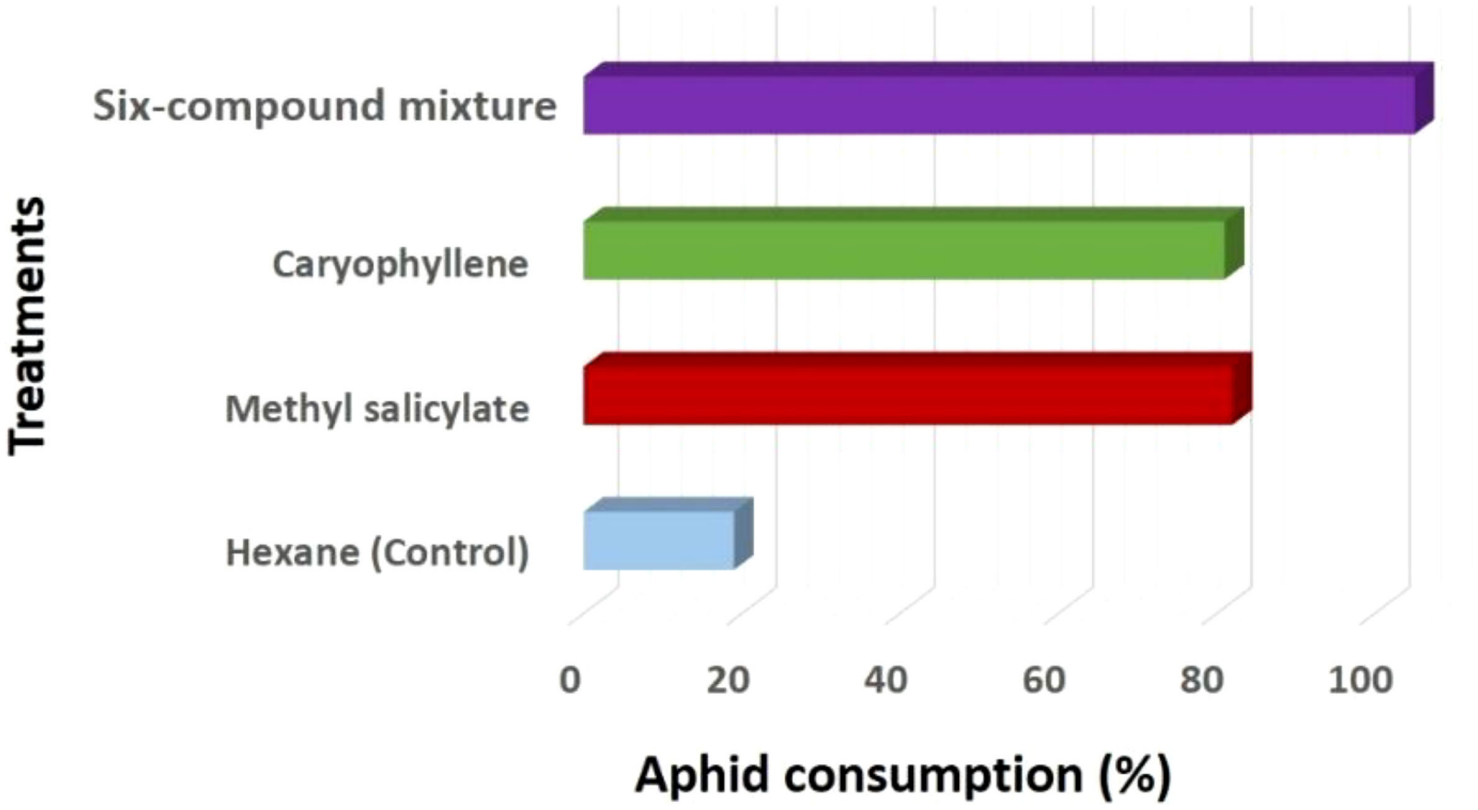
Variation in aphid consumption (%) by *Coccinella undecimpunctata* across volatile treatments: methyl salicylate, caryophyllene, six-compound mixture (6-HIPVs) and a control (n-hexane), calculated using Equation 2. Values are expressed as mean ± SD (N = 10; 20 insects per test).


The Attraction Index (AI) (**Figure 5**) was calculated using the formula:

(1) $$AI\;\left(\%\right)=\frac{\left(Tt-Tc\right)}{\left(Tt+Tc\right)}\times 100$$

where Tt is the time spent in the treatment zone and Tc is the time in the control zone. Aphid consumption (AC) (**Figure 6**) was calculated using the Abbott correction formula for untreated controls:

(2) $$AC\;\left(\%\right)=\frac{Ae}{10}\;\times 100$$

where Ae is the number of aphids eaten (maximum of 10). Each treatment was replicated 10 times using different individual ladybirds.

To evaluate statistical differences between volatile treatments, a one-way analysis of variance (ANOVA) was performed on the attraction index and aphid consumption values. The ANOVA tested for significant differences across the three treatment groups (methyl salicylate, β-caryophyllene, and 6-HIPVs). Significance was determined at a 95% confidence level (α = 0.05), and *post-hoc* comparisons were conducted when necessary to identify pairwise differences among treatments (**Figures 5**, **6**; **Table 3**).

Table 3One-way ANOVA summary for the attraction index (%) of *Coccinella undecimpunctata* in response to three volatile treatments: methyl salicylate, caryophyllene, six-compound mixture (6-HIPVs) and a control (n-hexane) (α = 0.05; N = 10; 20 insects per test).

**Table:** 

Groups	Count	Sum	Average	Variance		
Hexane (Control)	10	0	0	0		
Methyl salicylate	10	171.4744	17.14744	5.649404		
Caryophyllene	10	154.8693	15.48693	6.043698		
Six-compound mixture	10	247.2856	24.72856	2.491758		
ANOVA						
Source of variation	SS	df	MS	F	P-value	F crit
Between Groups	3227.549	3	1075.85	303.3797	1.34E-25	2.866266
Within Groups	127.6637	36	3.546215			
Total	3355.213	39				

Principal Component Analysis (PCA) was conducted to explore the multivariate behavioral responses of *C. undecimpunctata* to the different volatile treatments. The input variables included the Attraction Index (%) and Aphid Consumption (%), calculated for each replicate using Equations 1 and 2 across all treatment groups.

Prior to analysis, the variables were standardized using z-score normalization to eliminate scale-related biases. PCA was performed based on the covariance matrix, and the first two principal components (F1 and F2) were extracted to visualize behavioral variation across treatments. A PCA biplot was generated to identify clustering patterns and assess similarities or distinctions in responses between treatments.

**Figure 7:**
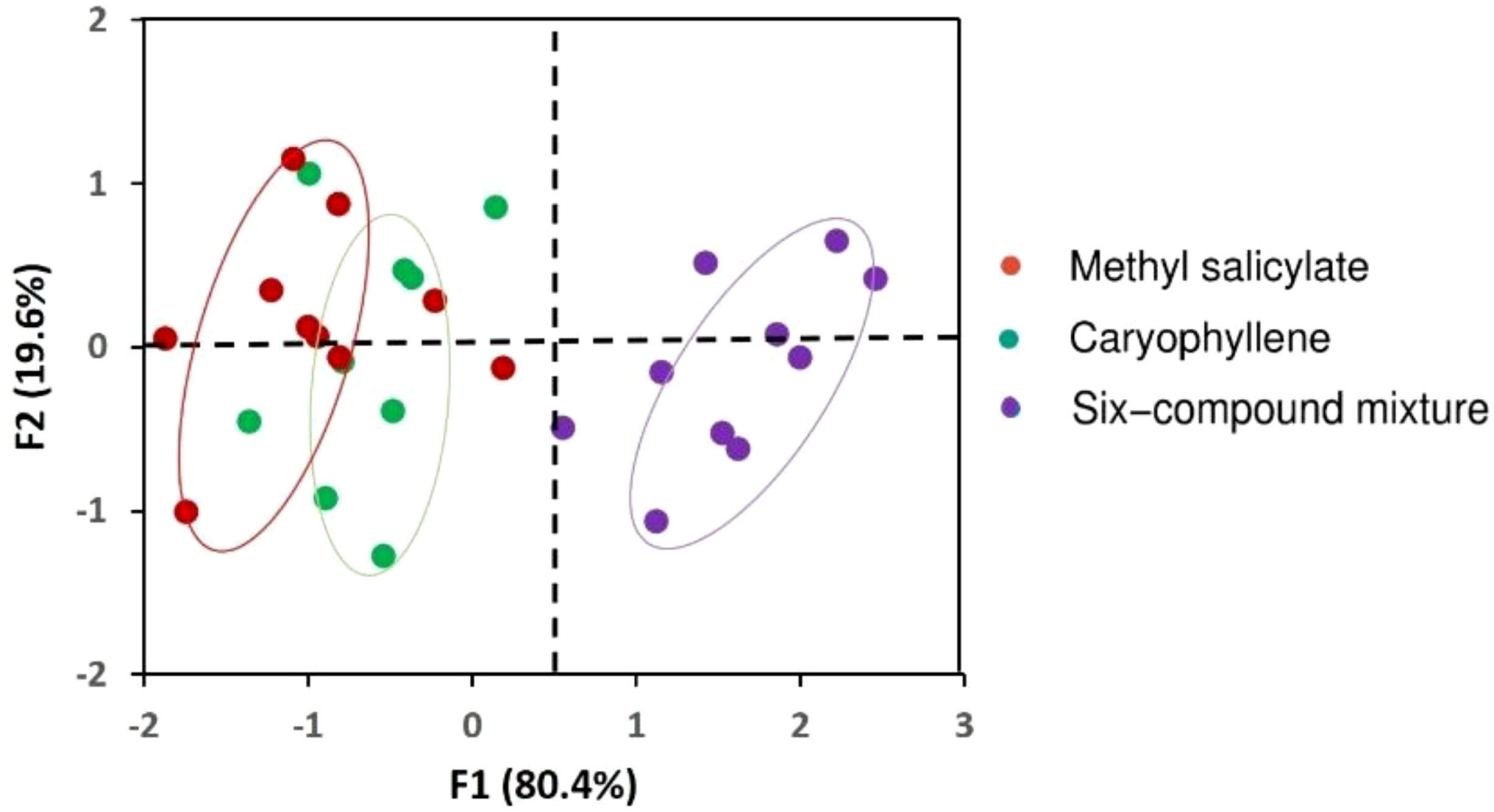
Principal Component Analysis (PCA) based on the attraction index (%) and aphid consumption (%) of *Coccinella undecimpunctata* across volatile treatments: methyl salicylate, caryophyllene, six-compound mixture (6-HIPVs). Correlation circle of the variables projected on axes F1 and F2.

All statistical analyses, including PCA, were performed using SRplot, an online data visualization platform (version 2023, PMID: 37943830, University of Nebraska-Lincoln, UNITED STATES) (Tang et al., 2023), with visual outputs generated to compare treatment effects (**Figure 7**).

## 3 Results

### 3.1 Overview of VOCs profiles in Pomegranates within ecological systems

In this study, we examined the VOCs emitted by pomegranate plants (*Punica granatum*) under various ecological interactions involving the pomegranate aphid (*A. punicae*), the ant species *T. magnum*, and the ladybird *C. undecimpunctata*.

Our investigation focused on two field-based ecological systems influenced by bottom-up processes. The first system included plant-aphid interactions over 24 hours (S1-DVB/twotrophic relationship: aphid-plant), followed by plant-aphid-ant interactions after introducing *T. magnum* at 24 hours (S2-DVB/tritrophic relationship: aphid-plant-ant). The second system examined plant-aphid-ant-ladybird interactions following the introduction of *T. magnum* and *C. undecimpunctata* at 24 and 48 hours (S3-DVB and S4-DVB, respectively).

These interactions among host plants, pests, protectors, and predators triggered the release of VOCs into the chemosphere. The VOCs were collected from plant branches using OLS and analyzed by GC-MS coupled with PCA.

#### 3.1.1 Triggering systemic acquired resistance in pomegranates using OLS systems-DVB

Significant differences were observed in the volatile profiles of AIP in the presence of predators and protectors. A total of 30 airborne metabolites were identified, influencing both beneficial and detrimental interactions within these ecological systems. Plants damaged by *A. punicae* altered their chemospheres, affecting the diversity and concentration of compound classes. Alkanes, aldehydes, esters, benzene derivatives, and terpenes were the most common airborne compounds released across treatments.

In the AIP (S1-DVB), the headspace contained benzene derivatives (53.44%), alkanes (43.62%), monoterpenes (5.07%), sesquiterpenes (5.14%), and alcohols (1.94%). After adding *T. magnum* (S2-DVB), the tripartite community’s headspace profile showed an increase in ketones (75.04%) and alkanes (19.84%), while monoterpenes, sesquiterpenes, and aldehydes were no longer detected. In the quadripartite community (S3-DVB), which included both *T. magnum* and *C. undecimpunctata*, there was a 12.92% reduction in alkanes and a complete absence of sesquiterpenes (**Table 2**; **Figures 2A, B**). Pomegranate plants damaged by *A. punicae* (n=15) demonstrated the ability to alter their volatile odor emissions. The presence of *T. magnum* reduced the number of VOCs to four (n=4), while the introduction of *C. undecimpunctata* increased the number of detected VOCs to 17 (n=17), compared to AIP (**Table 2**).

For non-targeted analysis, the chemosphere constituents of the AIP were primarily composed of o-xylene (bio #1), p-xylene (bio #2), 1-methyl-3-tert-butylbenzene (bio #5), m-ethylcumene (bio #6), 2-methylnaphthalene, 3-carene (bio #11), (+)-4-carene (bio #15), benzaldehyde (bio #16), 2,2,4-trimethyl-pentane (bio #18), tridecane (bio #21), caryophyllene (bio #27), and β-farnesene (bio #28). The alarm pheromone bio #28, benzenoids (bio #5, and #6), monoterpenes (bio #11, and #15), alkanes (bio #22, and #18), and sesquiterpene (bio #27), were uniquely identified in headspace of the G1–DVB and was absent in subsequent systems (G2-G4-DVB). While, p-xylene (bio #2) was the only compound detected in all four ecologic systems (**Table 2**; **Figures 2A, B**). Ester, ketone, or N-compounds were absent in the headspace of S1 but were detected in the other systems (**Table 2**; **Figures 2A, B**).

The addition of *T. magnum* significantly altered the chemosphere of AIP, with VOCs consisting of o-xylene (bio #1), p-xylene (bio #2), an unknown alkane-2 (bio #20), and with a dominance of 4-heptanone (bio #8). This ketone was classified as a primary ant pheromone. After the introduction of both *T. magnum* and *C. undecimpunctata*, the number of detected VOCs decreased to seven after 24 hours but increase to 17 after 48 hours of interaction, compared to the AIP airborne compounds, indicating dynamic changes in chemospheres over time. Following, 48 hours of ecological interaction, the chemosphere detected new VOCs, while others disappeared, resulting in a composition dominated by 99.73% of the identified compounds (**Table 2**, **Figures 2A, B**). These findings underscore the significant impact of ecological interactions on the VOC emissions of pomegranate plants, with specific compounds correlating strongly with the presence of particular species.

#### 3.1.2 Analysis of herbivore-induced plant volatiles

Across all samples collected at different stages of AIP interactions, several VOCs were consistently detected. Notably, β-farnesene was uniquely identified in HIPVs from AIP, suggesting its potential role in plant defense and aphid communication.

To gain a deeper understanding of the odor profile of pest-infested plants and to identify behaviorally active compounds that influence predator responses, employing complementary analytical methods is crucial. Our findings underscore the importance of integrating multiple techniques to accurately characterize these volatile signals and their ecological significance.

### 3.2 ANOVA analysis of VOC variability in ecological interactions

The ANOVA results (**Supplementary Table S1**) reveal that the biological compounds (rows factor) significantly impact the observed VOC variability. The F-statistic of 3.12 and an extremely low P-value (4.17e-07) indicate that the variability in VOCs is primarily driven by differences in the biological compounds, such as various benzene derivatives, alkanes, and terpenes (**Supplementary Tables S1**, **S2**). This suggests that specific compounds like o-Xylene, p-Xylene, and 4-Heptanone exhibit substantial variation across the interactions and drive the chemical profiles in the system (**Supplementary Table S2**).

In contrast, the experimental conditions (columns factor) did not significantly contribute to the VOC variability, as evidenced by the very low F-statistic (0.04) and a P-value close to 1 (0.999998881) (**Supplementary Table S1**). This implies that factors such as the different ecological interaction groups (G1: AIP, G2: AIP-T, G3: AIP-TC24, G4: AIP-TC48) did not substantially affect the VOC emissions.

The variance in VOCs across different biological compounds, with values ranging from low variance in compounds like alpha-Methylbenzeneacetaldehyde (0.0) to high variance in compounds such as 4-Heptanone (1124.5), further underscores the prominent role of biological compounds in shaping VOC emissions (**Supplementary Table S2**). Overall, the results highlight that the variability in VOC emissions is largely influenced by the biological compounds involved in the ecological interactions, while the experimental conditions play a negligible role in determining the chemical profiles.

### 3.3 Hierarchical clustering analysis of VOCs in pomegranate-aphid-ant-ladybird interactions

Hierarchical clustering analysis was employed to investigate the relationships among VOCs emitted during various ecological interactions. This approach aimed to elucidate how different biotic interactions influence the VOCs profiles of pomegranate plants. This study specifically examined the compositional and spatial proximity of individual VOCs across different ecological interactions. It focused on AIP, triplet interactions between AIP and *T. magnum* as well as quadruplet interactions involving AIP, *T. magnum*, and *C. undecimpunctata*. Pearson’s correlation was applied to compute distances between samples, enabling hierarchical clustering to visualize patterns of similarity and variation in VOC emissions across these interactions.

The relative abundance of each volatile compound was visualized using a color scale to highlight variations across the ecological systems. The resulting heat map provides a comprehensive depiction of VOC distribution, with each color corresponding to a specific compound. The scale ranges from red (maximum, 2) to orange (average, 0), and dark blue (minimum, –2), facilitating a clear interpretation of VOC fluctuations across various interaction scenarios.

To enhance the qualitative characterization of VOC relationships, we performed fingerprint analysis. In the topographic fingerprint, each column represents the signal peaks of a sample, while each row corresponds to a specific VOC across all samples. The color intensity reflects VOC concentration, with brighter colors indicating higher quantities. As illustrated in substantial variations in VOC content were observed across the four groups, underscoring distinct chemical signatures associated with each ecological interaction.

These findings suggest that hierarchical clustering analysis, combined with fingerprint visualization, effectively elucidates the impact of biotic interactions on VOC emissions in pomegranate plants. Such insights are crucial for understanding plant-insect dynamics and could inform strategies for pest management and ecological conservation.

### 3.4 Principal component analysis of VOC profiles in pomegranate-aphid-ant-ladybird interactions

To further investigate the variation in VOC emissions across different ecological interactions, PCA was performed. The PCA score plot (**Figures 4A, B**) revealed a clea separation of the ecological interaction systems into four distinct groups based on their VOC profiles, with significant differences (p< 0.05) between AIP alone and AIP in combination with *T. magnum* and *C. undecimpunctata.*

PCA of VOC profiles from the four study systems (S1-DVB, S2-DVB, S3-DVB, and S4-DVB) further confirmed the clear separation of ecological interaction groups. The first two principal components (F1 and F2) accounted for 73.8% of the total variability in the dataset, with F1 explaining 45.9% and F2 accounting for 27.9% of the observed variation (**Supplementary Table S3**; **Figure S1**).

F1 distinguished group G-4, which was enriched in 1-ethyl-3-methylbenzene (bio #3), 1,3,5-trimethylbenzene (bio #4), methyl salicylate (bio #10), sabinene (bio #12), limonene (bio #14), unknown alkane-3 (bio #22), pentadecane (bio #24), heptadecane (bio #25), 1-methyl-1H-imidazole (bio #26), and unknown-1 (bio #30). These compounds were predominantly located in the positive region of F1 (right side of the PCA), indicating a strong influence on this component.

F2 separated groups G-2 and G-3, which contained higher levels of methyl methanoate (bio #9), unknown alkane-2 (bio #20), and 4-heptanone (bio #8). These groups, represented by G2: AIP-T and G3: AIP-TC24, were positioned in the negative region of F2, suggesting their distinct chemical profiles.

Group G-1, identified by increased concentrations of 1-methyl-3-tert-butylbenzene (bio #5), m-ethylcumene (bio #6), 3-carene (bio #11), (+)-4-carene (bio #15), 2,2,4-trimethyl-pentane (bio #18), unknown alkane-3 (bio #22), and caryophyllene (bio #27), was located in the left positive region of F2, primarily represented by G1: AIP (**Supplementary Figure S1**).

Our analysis revealed that the presence of *C. undecimpunctata* notably influenced the separation of groups along the F1 axis, underscoring the ladybird’s significant role in modulating VOC emissions in the pomegranate-aphid-ant-ladybird system. The differentiation of the four ecological groups along both F1 and F2 axes highlights the intricate chemical coordination governing interactions among the quadrupletships. These findings shed light on the complexity of ecological relationships and chemical communication mechanisms, emphasizing the pivotal roles of specific VOCs in shaping plant responses to infestations and the presence of natural protectors.

**Figure 8:**
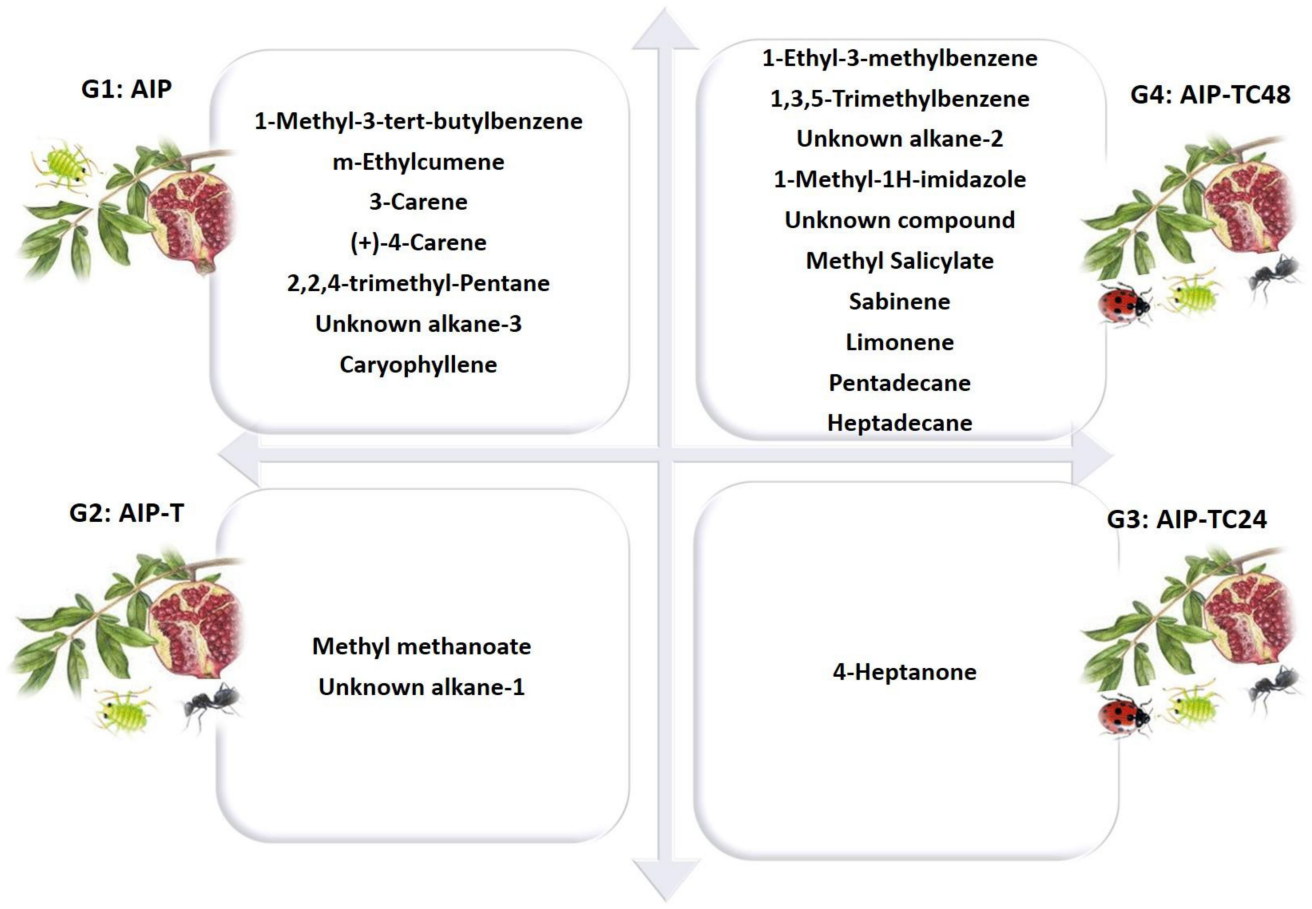
Specific biomarkers were detected from the AIP plant, AIP + Ant, AIP + Ant + Ladybird (24 hours), and AIP + Ant + Ladybird (48 hours) using OLS coupled with GC-MS and analyzed using PCA analysis. The 30 VOCs were reduced by PCA statistical analysis to 20 biomarkers (7 vs 15) for G1: AIP, (2 vs 4) for G2: AIP-T, (1 vs 7) for G3: AIP-TC24, and (10 vs 17) for G4: AIP-TC48.

The chemical and statistical analyses, as presented in **Table 1** and **Figures 4A, B**, demonstrate that VOC profiles can effectively distinguish among the four ecological communities, as illustrated in **Figure 8**. The observed variations in VOC composition and concentration across different interactions reinforce the influence of biotic factors, such as aphids, ants, and ladybirds, on pomegranate VOC emissions. The distinct chemical responses triggered by these species’ interactions suggest potential avenues for developing eco-friendly pest management strategies that leverage natural ecological processes.

### 3.5 Impact of Key Plant Biomarkers on the Behavior of *Coccinella undecimpunctata*

The One-Way ANOVA analysis was conducted to compare the Attraction Index (%) of *C. undecimpunctata* in response to four volatile treatments: Hexane (Control), Methyl salicylate, Caryophyllene, and 6-HIPVs. The data for each treatment revealed the following: for the hexane (Control) group, the attraction index remained at 0.00 across all 10 replicates, indicating no attraction. The methyl salicylate treatment had an average attraction index of 17.15, with values ranging from 12.45 to 21.52. The caryophyllene treatment showed an average attraction index of 15.49, with values ranging from 11.86 to 20.16. Finally, the 6-HIPVs mixture treatment yielded the highest average attraction index of 24.73, with values ranging from 22.10 to 26.42.

The results from the One-Way ANOVA (**Table 3**) indicated a highly significant difference between the groups. The Between Groups Sum of Squares (SS) was 3227.55, and the Within Groups SS was 127.66. The calculated F-value was 303.38, which far exceeded the critical F-value of 2.87 at an alpha level of 0.05. Furthermore, the P-value was found to be extremely small (1.34E-25), suggesting that the observed differences between groups are statistically significant. Since the P-value is less than 0.05, the null hypothesis is rejected, confirming that there is a significant difference in the attraction index among the four volatile treatments.

In addition to attraction behavior, aphid consumption by *C. undecimpunctata* was also assessed under the same volatile treatments. The hexane (Control) group exhibited the lowest aphid consumption, with values ranging from 10 to 30 and an average of 19 aphids consumed. In contrast, the 6-HIPVs group showed the highest predation, with consumption values ranging from 90 to 120 and an average of 105 aphids consumed. Methyl salicylate and caryophyllene treatments also led to elevated predation levels, with mean values of 82 and 81 aphids consumed, respectively. These results suggest that exposure to specific volatile compounds, particularly complex mixtures, not only increases attraction but also enhances predation behavior in *C. undecimpunctata* (**Figures 5**, **6**; **Table 3**).

To further support and visualize the patterns observed in the ANOVA, a Principal Component Analysis (PCA) was performed. The first two principal components explained 100% of the total variance, with F1 accounting for 80.4% and F2 for 19.6% (**Figure 7**). F1 was primarily associated with high attraction and aphid consumption, particularly in response to the 6-HIPVs mixture, while F2 differentiated responses to individual compounds such as methyl salicylate and caryophyllene. The PCA biplot revealed the formation of three distinct clusters, each corresponding to a different response group. The mixture treatment formed a clearly separated cluster, distinctly positioned from the groups corresponding to the individual volatiles and the control. This separation highlights the strong positive association between the mixture and predator activity, suggesting additive or synergistic effects of combined volatiles. Therefore, the PCA confirms the ANOVA results by demonstrating that *C. undecimpunctata* exhibits significantly different behavioral responses depending on the volatile treatment, with the mixture standing out as a key factor influencing both attraction and predation.

## 4 Discussion

Biological control research has increasingly emphasized the role of chemical cues in insect-plant interactions, particularly plant-emitted infochemicals that guide pests to suitable host plants (Conti et al., 2021; Vargas et al., 2023). In recent years, semiochemical-based strategies have emerged as effective tools for manipulating insect behavior and improving crop pest management (Conti et al., 2021). However, ecological interactions across multiple trophic levels remain highly complex. Cascading effects within these systems spanning three or more trophic levels are influenced by both bottom-up (resource availability) and top-down (natural enemy pressure) mechanism (Senft et al., 2019). Due to their intricacy, these interactions are often studied in isolation, leaving the combined strength of their effects largely unexplored (Senft et al., 2019). Pomegranate responses to environmental stress and biotic interactions remain complex and not fully understood. The volatile profile of a system can undergo significant changes due to biotic factors, such as the introduction or removal of organisms including symbionts, and abiotic factors, including temperature, pH, humidity, light exposure, geographical location, and seasonal variations (Takabayashi et al., 1994). This study advances our understanding of aphid chemical ecology and its interactions with the host plant (pomegranate), natural enemy (ladybird), and protector (ant), offering insights for eco-friendly pest management. By analyzing volatile shifts, we identify key compounds for pest trapping and repellence, utilizing plant defense signals, aphid alarm pheromones, and ladybird cues to enhance biological control strategies.

### 4.1 Exploitation of chemical communication

Pest infestation, like other ecological interactions, significantly alters the volatile profiles emitted by host plants, thereby influencing pest behavior (Conchou et al., 2019; Kutty and Mishra, 2023). Research has demonstrated that secondary VOCs emissions triggered by pest attacks plays a critical role in plant-insect interactions, either attracting or repelling insects as part of their evolutionary dynamics (Ehrlich and Raven, 1964; Giunti et al., 2018). Other studies also indicate that VOC alterations are closely associated with the specific pest species involved (H. G. Guo et al., 2023). Similarly, our study highlights the ecological interaction within the pomegranate–aphid–ant–ladybird system, where the balance between ecological dynamics and chemical signaling is essential for ecosystem stability and function. We found that ecological interaction systems notably modify the chemosphere of AIP, particularly in the presence of protective species and when both protectors and enemies coexist (**Supplementary Figure S1**; **Table 2**; **Figure 2B**).

### 4.2 Ecological relevance of terpenoid production

Uninfected pomegranate plants naturally produce terpenes, the primary bioactive compounds in essential oils (Hosseini et al., 2021),which play several ecological roles, including defense against pathogens and herbivores, pollinator attraction, inter-plant communication, and root competition (K. J. Damodaram et al., 2014; Hosseini et al., 2021). Terpenoids exhibit eco-friendly insecticidal, herbicidal, fungicidal, and bactericidal properties (Jung, 2014). However, pomegranates contain only small amounts of monoterpenes (Maphetu et al., 2022; Marsoul et al., 2020), including alpha-terpinene, alpha-terpineol, and 3-carene (bio #11) (D. Wang et al., 2018). Our findings support these observations, showing that only 5.07% of the monoterpenes in headspace of AIP consisted of 3-carene (bio #11) and (+)-4-carene (bio #15). Previous studies has demonstrated that the odor of pomegranate leaves and flowers primarily originates from sesquiterpene hydrocarbons, including β-caryophyllene, β-farnesene, and trans-α-bergamotene (Guler and Gül, 2016), with α-humulene being the dominant compound in the stem and leaves. Additionally, the VOCs of uninfected pomegranates is dominated by non-terpenes, particularly aldehydes and esters (Bandeira Reidel et al., 2018). The AIP profile showed a higher concentration of aldehydes (20.47%) and benzenoids (53.44%), while esters were nearly absent (**Table 2**, **Figures 2a, b**). These compounds valuable in AIP insights into the plant’s status (K. J. P. Damodaram et al., 2014; Piesik et al., 2013).

Among the VOCs identified, β-caryophyllene (bio #27) was the most abundant terpenoid in AIP headspace. This sesquiterpene has been detected throughout various phenological stages of pomegranates (Hosseini et al., 2021; Melgarejo et al., 2011) and plays a significant role in ecological interactions, acting as a pheromone, allomone, or kairomone (Tavera et al., 2023a). While insect can synthesize β-caryophyllene (bio #27), plants generally release it naturally or in response to herbivore attacks. Research has demonstrated its function as a kairomone for various insect species, such as *Aphis fabae* (Hemiptera) (Webster et al., 2008), *Helopeltis bakeri* (Tavera et al., 2023a), *Myzus persicae* (Homoptera) (Beale et al., 2006), and *Bruchophagus roddi* (Hymenoptera) (Kamm and Buttery, 1986). In coffee systems, β-caryophyllene (bio #27) application resulted infestations of *Hypothenemus hampei* by 33–45%, suggesting its potential role in pest management strategies (Kansman et al., 2023; Sadeh et al., 2017). However, its effects on natural enemies vary. While limonene has shown attractant and repellent properties depending on concentration, β-caryophyllene consistently displayed repellency (Kansman et al., 2023), potentially deterring beneficial predators at high levels (Sadeh et al., 2017). In contrast, β-farnesene (bio #28) plays a more direct role in mediating trophic interactions between plants, aphids, and their natural enemies. Aphids secrete β-farnesene (bio #28) as an alarm pheromone (B. Wang et al., 2022), yet we did not detect in the AIP + An + Lb samples (AIP-TC24 and AIP-TC48). Additionally, AIP produces β-farnesene (bio #28) as a semiochemical to attract natural enemies (Vandermoten et al., 2012; B. Wang et al., 2022), although it accounted for only 0.05% of the VOCs (**Table 2**). The near absence of β-farnesene (bio #28) in the AIP-TC24 and AIP-TC48 treatments aligns with previous research showing that aphids under predator attack release only minimal amounts of this compound (Vosteen et al., 2016). This raises questions about β-farnesene’s (bio #28) reliability as a kairomone for aphid predators, particularly when other VOC signals are present.

We hypothesize that aphid-derived β-farnesene (bio #28) alone may not be sufficient to attract natural enemies, as they likely rely on a more complex blend of herbivore-induced plant volatiles (HIPVs) to detect AIP. Supporting this, in treatments where *C. undecimpunctata* interacted with AIP (AIP-TC24 and AIP-TC48), we observed the emission of unique VOCs linked to aphid enemies, including tricyclene, limonene, and various alkanes (unknown alkane-2, tridecane, pentadecane, and heptadecane) (**Table 2**, **Figure 8**). These compounds were also identified in the study by Alotaibi et al. (2023), which characterized aphid species based on mechanical stress-induced chemical profiles. Specifically, *A. punicae* was distinguished by a unique VOC signature composed of esters, benzenoids, and monoterpenes.

This integrated perspective suggests that β-farnesene (bio #28) and β-caryophyllene (bio #27) contribute differently to pest control strategies. While β-farnesene (bio #28) may function primarily as an aphid alarm pheromone, it appears insufficient alone to attract natural predators, necessitating other plant-emitted cues. On the other hand, β-caryophyllene (bio #27), despite its potential in deterring pests, may also repel beneficial insects at certain concentrations. Understanding how these VOCs interact within the pomegranate-aphid-ant-ladybird system is crucial for developing targeted bio-insecticide strategies. Specifically, future research should explore the potential of manipulating HIPVs to enhance the attractiveness of pomegranate VOCs to aphid predators, thereby improving biological control in agricultural ecosystems.

### 4.3 Primary diagnostic for AIP

Research on VOC emissions in pomegranate plants in response to insect infestations remains limited. However, a previous study reported changes in volatile profiles following *Ectomyelois ceratoniae* infestation (Sahraeian et al., 2021). Utilizing plant-emitted VOCs to influence insect behavior presents a promising strategy for pest management, potentially reducing pesticide use and minimizing environmental and human health risks. In this study, we collected and analyzed VOC emissions from AIP trees under four different scenarios: AIP (S1-DVB) alone, AIP with ants (S2-DVB), and AIP with both ants and ladybirds after 24 and 48 h (S3-DVB and S4-DVB).

The distribution of non-targeted VOCs was significantly influenced by the ecological interaction systems. Notably, the release of terpenes was strongly affected by the progression of AIP (S1-DVB) and the introduction of *C. undecimpunctata* (S3-DVB-24 and S4-DVB-48). Terpenes, particularly sesquiterpenes like caryophyllene (bio #27) and β-farnesene (bio #28), are known in respond to aphid feeding, attracting predator. At higher concentrations, these compounds can even repel aphids (Warneys et al., 2018). β-Farnesene (bio #28) is a common component in the essential oils pomegranate (Bandeira Reidel et al., 2018; Zhaleh et al., 2023), and confirmed that the absence of herbivore-induced feeding significantly produce sesquiterpenes at a low rate (Kivimäenpää et al., 2016), but its presence decrease or increase their production (Kivimäenpää et al., 2020, 2016; Mäntylä et al., 2017).

In our system, we observed a low level of β-farnesene (bio #28) and no detectable methyl salicylate (MeSA) (bio #10) in the AIP treatment (S1-DVB), while caryophyllene (bio #27) was significantly elevated (**Table 2**). These findings align with previous research (Kivimäenpää et al., 2020), which indicated that β-farnesene (bio #28) and MeSA (bio #10) generally respond to aphid infestation after approximately 10 days, though with minimal increases. In contrast, caryophyllene emissions increased dramatically by a factor of 27 immediately following aphid infestation (Kivimäenpää et al., 2020). These results suggest that the aphid infestation in this study was still in its early stages, characterized by low β-farnesene (bio #28) levels and high caryophyllene (bio #27) concentrations, with no detectable MeSA (bio #10).

Our study emphasizes the importance of early detection of pest and pathogen infestations in pest management (Li et al., 2019). These findings also suggest that VOCs, particularly caryophyllene (bio #27), may serve as an early indicator for aphid infestations, which could be valuable for integrated pest management (IPM) strategies. However, to further improve the study’s relevance and impact, future research should incorporate more rigorous statistical validation and explore the practical application of these findings in real-world agricultural scenarios.

### 4.4 How statistical analysis can aid integrated pest management research?

#### 4.4.1 Comparison of ANOVA, PCA, and heatmap results

The ANOVA, PCA, and heatmap results together provide comprehensive insights into the factors driving VOC variability across ecological interactions. ANOVA revealed that biological compounds significantly influenced VOC profiles (P-value: 4.17e-07), while experimental conditions had minimal impact (P-value: 0.9999989) (Küntzel et al., 2016). These findings align with PCA, where distinct separation of groups (G1-G4) was observed based on their VOC composition. Notably, the first two principal components (F1 and F2) explained 73.8% of the total variance, with groups showing clear differentiation based on the presence of species such as *T. magnum* and *C. undecimpunctata*. Group G1, which represented the AIP alone system, exhibited a unique VOC profile, characterized by compounds like 1-methyl-3-tert-butylbenzene (bio #5) and caryophyllene. In contrast, groups G2 and G3, which involved AIP with *T. magnum* and AIP with both *T. magnum* and *C. undecimpunctata*, displayed distinct chemical compositions.

The heatmap further corroborated these results by visually illustrating the dynamic changes in VOC emissions over time. It showed that specific compounds like 4-heptanone and methyl methanoate were strongly associated with certain ecological interactions. The heatmap also highlighted the temporal nature of VOC profiles, with certain compounds appearing or disappearing based on the presence of different species, reinforcing the idea that biological factors, rather than experimental conditions, drive VOC changes.

In conclusion, the integration of ANOVA, PCA, and heatmap analyses underscores the dominant role of biotic factors in shaping VOC emissions, emphasizing the complex and dynamic interactions among species in the pomegranate ecosystem (Y. Guo et al., 2019). This highlights the significant influence of biological compounds, particularly the presence of natural predators and protectors, in shaping the chemical communication of pomegranate plants.

#### 4.4.2 Investigating VOC profiles in AIP: implications for integrated pest management strategies

The oligophagous insect *A. punicae* is a major pest of pomegranates, infesting young leaves, shoots, flowers, buds, and developing fruit (Pathania et al., 2019). Our previous study identified *A. punicae* as highly prevalent in the Taif Governorate, where it preferentially colonizes pomegranate plants (Alotaibi et al., 2023). Early detection of plant pathogens and insect pests is crucial for identifying VOC biomarkers that could serve as bioinsecticides in IPM. This study, examines VOC profiles in a four-way ecological interaction involving AIP, their natural enemies (*C. undecimpunctata*), and mutualistic protectors (*T. magnum*). Using hierarchical clustering analysis (HCA) and principal component analysis (PCA), we identified distinct VOC patterns, which are visualized in a heat map.

HCA results revealed significant variation in VOC emissions across ecological treatments. The heat map showed that the quadruplet interaction after 48 hours, G4 (AIP-TC48), had the most distinct VOC profile, with 17 VOCs among the 30 detected, peaking at 48 hours when the host plant interacted with both the predator (*C. undecimpunctata*) and protector (*T. magnum*). In contrast, AIP alone (G1: AIP), which exhibited 15 VOCs, was not clustered with any other system, suggesting a unique VOC signature in the absence of ecological interactions.

With the presence of *T. magnum* and *C. undecimpunctata* influenced aphid movement and VOC emissions. However, treatments with *T. magnum* alone and with both *T. magnum* and *C. undecimpunctata* at 24 hours (G2: AIP-T and G3: AIP-TC24, respectively) exhibited lower VOCs diversity (4 and 7 VOCs, respectively) and reduced chemical abundance (blue color), whereas AIP alone (G1: AIP) had the highest VOC diversity and intensity (red color).

The introduction of *T. magnum* appeared to suppress aphid dispersal, as its semiochemicals are known to inhibit aphid movement, disrupt wing development (Oliver et al., 2007; Thurman et al., 2019), and alter aphid physiology (Oliver et al., 2007). Consequently, *T. magnum* led to an increase in local aphid density and honeydew availability but a reduction in overall VOC emissions, likely due to decreased aphid mobility. These semiochemicals could be exploited in IPM by limiting aphid spread through behavior-modifying compounds.

The chemical signals exchanged between *T. magnum* and aphids serve as critical communication channels in maintaining their mutualism (Xu and Chen, 2021). However, the dual role of ants in agroecosystems presents both risks and benefits for IPM. While they can enhance soil quality and suppress certain plant pathogens (Schifani et al., 2024), their protection of aphids can counteract the effectiveness of natural enemies. Understanding these dynamics is essential for developing IPM strategies that reduce pest populations without disrupting beneficial ant-mediated ecological services.

At 48 hours, the quadruplet interaction between AIP, *T. magnum*, and *C. undecimpunctata* resulted in a VOC increase, potentially indicating an evolved response to predation pressure (Oliver et al., 2007). This suggests that *T. magnum* semiochemicals may function as protective signals, modulating aphid behavior and influencing chemical communication within the ecosystem. The elevated VOC concentrations at this stage likely play multiple ecological roles, including aiding natural enemies in locating prey, repelling competing herbivores, and facilitating plant-plant signaling, all of which are valuable for IPM implementation (Bezerra et al., 2021).

These findings highlight the potential of VOCs as bioindicators for pest presence and suggest their application in developing eco-friendly pest management strategies. Specifically, VOCs can be leveraged for: (1) early pest detection through VOC-based monitoring tools, (2) attracting natural enemies by mimicking predator-associated VOCs, and (3) repelling aphids by utilizing deterrent compounds identified in pomegranate emissions.

Further statistical analyses, such as PCA, should be conducted to identify key VOC biomarkers for future validation through field trials and bioassay analysis. Bioassays will help determine the behavioral responses of aphids, natural enemies, and other associated organisms to specific VOCs, providing crucial insights into their role in attraction, repellency, or predator-prey interactions. This integrated approach will facilitate the practical implementation of VOC-based monitoring, semiochemical disruption, and natural enemy enhancement in IPM programs.

### 4.5 Quadruplet interaction-derived repellent and attractant substances for use in IPM

The application of a specific ecological statistic tool (**Figures 4A–C**) enhanced our understanding of the complex relationships that occur during pomegranate infestation by *A. punicae*. This approach facilitates the non-destructive early-stage diagnosis of aphid infestations. Furthermore, it aids entomologists in chemically tracking infestation status with precision by detecting significant biomarkers identified through PCA analysis (**Figures 4A,B**), as further explained in **Figure 8**. Focusing on key biomarkers in each chemosphere and analyzing variations in their emission profiles can significantly benefit aphid management strategies.

PCA separated the headspace profiles of the study systems into four distinct groups. The first group (G1), representing the AIP system (G1: AIP), contained seven key volatile compounds: 1-methyl-3-tert-butylbenzene (bio #5), m-ethylcumene (bio #6), 3-carene (bio #11), (+)-4-carene (bio #15), 2,2,4-trimethyl-pentane (bio #18), unknown alkane-3 (bio #22), and caryophyllene (bio #27). These compounds were identified as the primary volatile biomarkers with a strong repellent effect on *A. punicae*, directly influencing insect behavior. The introduction of *T. magnum* altered the interaction between aphids and host plants. As shown in **Figure 8**, the volatile profile of G2: AIP-T, which includes methyl methanoate (bio #9) and an unknown alkane-1 (bio #19), reflects the integration of *T. magnum* into the AIP system. This ester (bio #9) is widely recognized for its role in plant protection and insecticide formulations (Kaiser et al., 2021). It is also used as a fumigant and larvicide in pest control within agriculture and IPM practices (Haritos et al., 2006; Stejskal et al., 2021). In the presence of both *T. magnum* and *C. undecimpunctata* after 24 hours, the volatile profile pattern of G3: AIP-TC24 was characterized exclusively by the 4-heptanone biomarker (bio #8). This ketone biomarker, previously identified in our study, was detected in the profile of *A. punicae* and is associated with its symbiotic ant, *T. magnum* (Alotaibi et al., 2023). The high variability of this compound in the headspace may be linked to ant activity, particularly that of *Azteca* ants (Adams et al., 2020; Mayer et al., 2021). Numerous studies have identified bio #8 ketone biomarker as an alarm pheromone in ants, particularly within the *Azteca* genus. Some researchers suggest that this VOC may act as an anesthetic for harmful insects (Mayer et al., 2021; Papachristoforou et al., 2012). The chemosphere of *Azteca* ant nests includes 6-methyl-5-hepten-2-one, a known defense pheromone, reinforcing the role of 4-heptanone as a common alarm pheromone secreted by many ant species (Francelino et al., 2006; Jardine et al., 2020; Olubajo et al., 1980). It appears that ladybird’s predation aphid triggers *Azteca* ants to emit alarm pheromones outside in G3: AIP-TC24. This defensive response may serve to repel ladybirds (**Figure 8**). The chemical profiles of the quadruplet ecological interactions between AIP, *T. magnum*, and *C. undecimpunctata* after 48 hours (G4: AIP-TC48) were clearly defined, with the sample scores predominantly distributed along the first F1 axis (45.9% of the total variance; **Figures 4A, B**). This axis was strongly associated with the concentrations of several key compounds that were exclusive to the interaction between ladybirds and aphids. Among these biomarkers, we identified benzenoids (1-ethyl-3-methylbenzene (bio #3) and 1,3,5-trimethylbenzene (bio #3)), N-compound (1-methyl-1H-imidazole (bio #26)), alkane (unknown alkane-2 (bio #20), specific to aphid cuticles), and monoterpenes (sabinene (bio #12) and limonene (bio #12)). These compounds were previously identified in our study as pheromones induced by aphid feeding (Alotaibi et al., 2023).

In this complex profile, methyl salicylate (MeSA), pentadecane, heptadecane, and sabinene were also detected, though they played less prominent roles. Previous studies have demonstrated that MeSA emitted by strawberries attracted ladybirds to host plants, and similar responses have been observed in other predatory insects, including adult hoverflies and green lacewings, when exposed to MeSA in cranberry systems (Rodriguez-Saona et al., 2011; Salamanca et al., 2019). Additionally, certain alkanes emitted by infested green leaves have been classified as beneficial hydrocarbons due to their kairomone activity for attracting carnivorous insects, while pentadecane and heptadecane were identified as detrimental hydrocarbons (Paul et al., 2002).

**Figure 9:**
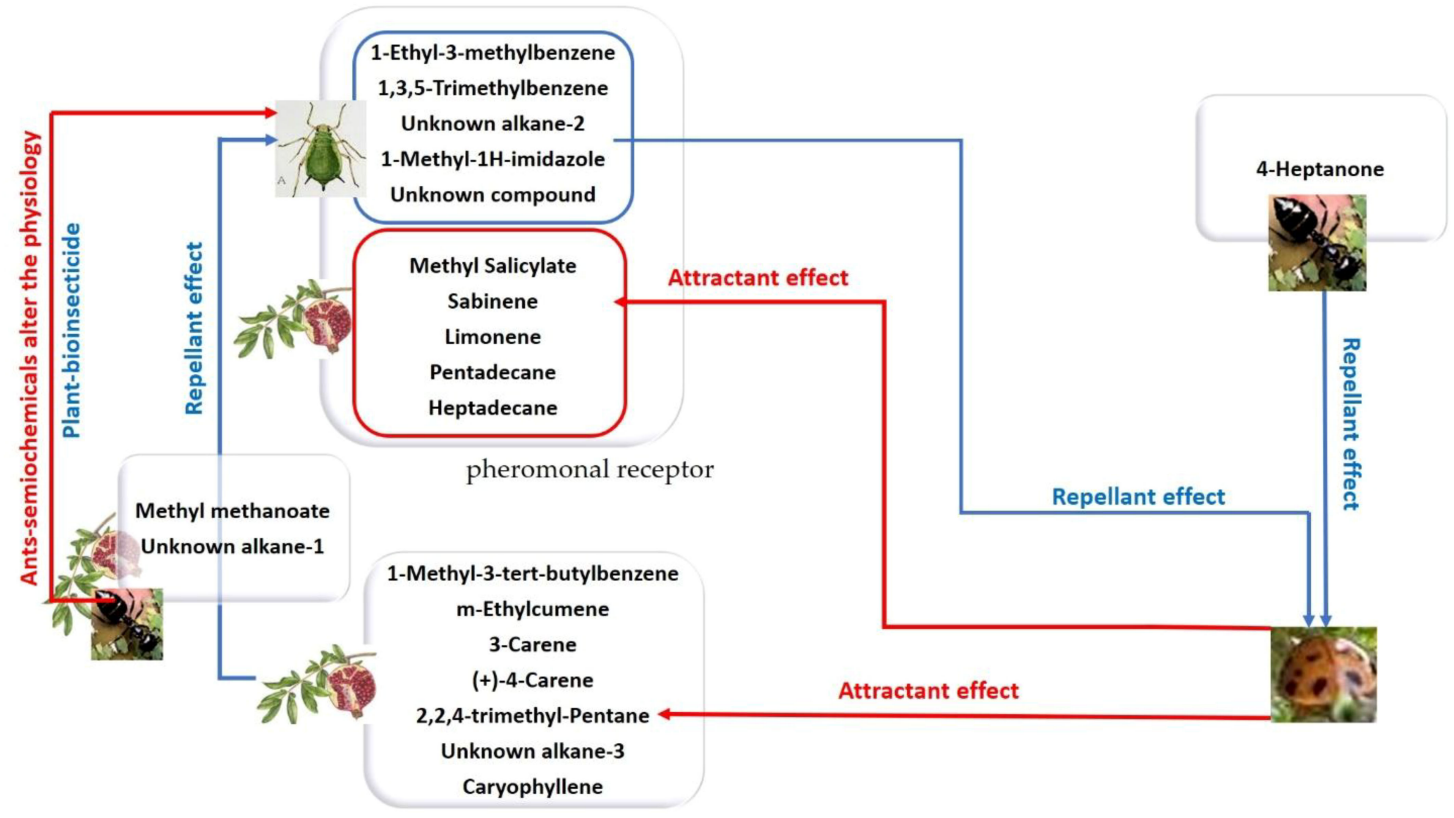
A diagram illustrating the impact of a pattern biomarker obtained through GC-MS and PCA analysis.

Our findings also revealed fluctuating interest in certain biomarkers. Notably, monoterpenes such as limonene and sabinene, which were absent in the headspace of AIP alone, became detectable after interaction with *C. undecimpunctata*. Although literature reports varying effects, some studies suggest that sabinene and limonene increase in plants infested with aphids, potentially contributing to their repellent properties (Ahmed et al., 2022; Dieudonné et al., 2022). The biomarker pattern identified in the quadruple ecological interaction between AIP, *T. magnum*, and *C. undecimpunctata* after 48 hours formed the basis for two distinct groups (**Figure 9**). The first group: aphid-infested host plants-derived compounds, which play a role in attracting *C. undecimpunctata*, while the second group: aphids-derived compounds, which contribute to the repellent effect on natural enemies (**Figure 9**).

These findings suggest that key VOCs emitted during ecological interactions could be leveraged for the development of eco-friendly pest management strategies. By identifying attractant and repellent VOCs, IPM programs can be optimized to enhance natural enemy efficacy while minimizing aphid infestations.

### 4.6 Influence of natural plant biomarkers from plant-ant-aphid-ladybird interactions on *Coccinella undecimpunctata* attraction and predation

The results from the analysis indicate that the three volatile treatments significantly influenced the attraction index of *Coccinella undecimpunctata*. The 6-HIPVs mixture treatment produced the highest average attraction index, suggesting that combined volatile compounds are more attractive to the ladybird than individual components. The methyl salicylate and β-caryophyllene treatments also induced significant attraction, but they were less effective than the mixture. As expected, the hexane (Control) group showed no attraction.

These findings align with previous studies demonstrating that complex blends of volatile compounds can elicit stronger behavioral responses in insects. For instance, methyl salicylate has been shown to be a potent attractant for ladybird such as *Coccinella septempunctata*, triggering electrophysiological responses and increasing field captures (Gençer et al., 2017; Zhu and Park, 2005). Similarly, β-caryophyllene has been identified from aphid-infested plants as attractive to predatory ladybird, especially when combined with other plant volatiles like methyl salicylate (Tavera et al., 2023a; Yi et al., 2023). The higher attraction observed in the 6-HIPVs mixture treatment in this study could be attributed to synergistic interactions among the compounds, enhancing the overall attractiveness.

Crucially, the 6-HIPVs compounds used in the mixture were identified through a combination of ecological observations and statistical analysis (Principal Component Analysis, PCA), focusing on the interaction between the host plant, aphids, tending ants, and *C. undecimpunctata* (**Figure 9**). The selected compounds represent key biomarkers associated with multi-trophic signaling in this system, highlighting the complex relationships among plants, herbivores, and natural enemies. Furthermore, through non-targeted analysis, we detected a specific key biomarker that may play a critical role in attracting ladybirds. This biomarker holds significant potential for use in IPM strategies, particularly in the agricultural context of the Taif governorate, where *C. undecimpunctata* is a valuable biological control agent.

Importantly, the PCA confirmed the behavioral patterns observed in the ANOVA. The first two principal components (F1 and F2) explained 100% of the total variance, with F1 accounting for 80.4% and F2 for 19.6%. F1 was strongly associated with both high attraction and predation activity, particularly in response to the mixture treatment. F2 helped differentiate responses to the individual compounds, methyl salicylate and β-caryophyllene. The PCA biplot revealed the formation of three distinct groups: two partially overlapping clusters representing the individual compounds (β-caryophyllene and methyl salicylate), and a third, clearly separated cluster for the mixture treatment, which included β-caryophyllene and methyl salicylate as well as sabinene, limonene, pentadecane, and heptadecane. This clear spatial separation of the mixture confirms its unique effect on *C. undecimpunctata* behavior and suggests additive or synergistic interactions among the volatiles, consistent with findings from similar studies (Ali et al., 2023; Turlings and Erb, 2018). PCA therefore strengthens the conclusion that plant volatile complexity plays a critical role in enhancing predator attraction and activity.

One possible hypothesis to explain our results is that the presence of certain compounds in the environment may influence the attraction of *C. undecimpunctata* to volatile treatments. It has been suggested that specific volatiles could inhibit the attraction of ladybirds. For instance, compounds such as β-caryophyllene, even though they attract predatory ladybirds when used in isolation, may interfere with the effectiveness of other plant volatiles in a mixture. (B. Wang et al., 2025). This could explain why, in our study, the 6-HIPVs mixture treatment yielded the highest attraction index, while individual compounds such as methyl salicylate and β-caryophyllene showed relatively weaker responses.

A potential mechanism underlying our findings could be the competitive or synergistic interactions between volatile compounds. Similar to how certain plant volatiles can diminish the efficacy of (E)-β-farnesene in repelling aphids such as β-caryophyllene in the case of hop aphids, comparable interactions may influence ladybird attraction (B. Wang et al., 2025). These dynamics could explain why *C. undecimpunctata* exhibited a stronger attraction to the 6-HIPVs mixture, where synergistic effects among the volatiles may amplify the behavioral response. In contrast, individual compounds presented in isolation may lack the complexity needed to elicit a comparable level of attraction.

Moreover, the continuous exposure of *C. undecimpunctata* to volatile treatments could lead to habituation, as seen in other species. If ladybirds are exposed to the same volatile blend over extended periods, it’s possible that their attraction response may diminish, which is a phenomenon seen in other pest management systems where insects habituate to continuous volatile exposure (Tewari et al., 2014; Zhu and Park, 2005).

Thus, the combination of methyl salicylate, β-caryophyllene, and other compounds in the mixture might work together to enhance the overall attractiveness to *C. undecimpunctata*. However, the presence of additional volatiles in the environment could modify or diminish this response, underscoring the complexity of using volatile-based attractants in IPM strategies.

This synergistic action is supported by earlier findings, where combinations of methyl salicylate, β-caryophyllene, and other green leaf volatiles (e.g., cis-3-hexenol) significantly increased attraction responses in *Coccinella s*eptempunctata and *Coccinella* transversalis under olfactometer assays (M. Ali et al., 2023; M. A. Ali et al., 2023). Conversely, the lower response to individual compounds like methyl salicylate and β-caryophyllene in our study suggests that specific volatile blends may be necessary to maximize attraction, which is critical for the development of effective biological control strategies.

The extremely low P-value (1.34E-25) offers strong evidence that the observed differences are not due to random variation. These results underline the potential for the development of more effective volatile-based attractants for *C. undecimpunctata*, which is widely used in IPM programs. Future studies should investigate the specific interactions and ratios between compounds within mixtures to better understand the chemical cues that drive behavioral responses in beneficial insects, enabling more targeted and effective pest control strategies.

In future studies, we also aim to investigate the biomarker identified in this study, particularly the compounds 1-ethyl-3-methylbenzene, 1,3,5-trimethylbenzene, and 1-methyl-1H-imidazole, which became dominant under the tetratrophic interaction (G4-AIP-TC48) (**Figures 8**, **9**) and are likely produced by the plant as part of aphid-induced chemical signaling. These volatiles may play a key role in aphid repellency and hold promising potential for enhancing targeted strategies in integrated pest management (IPM).

## 5 Conclusion and translational outlook

This study presents the first non-targeted metabolomic profiling of volatile organic compounds (VOCs) emitted by *Punica granatum* in response to *A. punicae* infestation and multitrophic interactions involving *T. magnum* and *C. undecimpunctata*. We identified scenario-specific VOC signatures, particularly caryophyllene, methyl salicylate, sabinene, limonene, pentadecane, and heptadecane, that act as biochemical indicators of herbivory and as semiochemical cues for natural enemies.

Behavioral assays demonstrated that a formulation blend of six herbivore-induced plant volatiles (6-HIPVs) significantly increased the attraction of *C. undecimpunctata*, supporting the role of synergistic volatile interactions in enhancing biological control efficacy. These findings emphasize the need to move beyond individual compounds and toward optimized mixtures for use in integrated pest management (IPM).

To enable practical field application, we propose the development of VOC-based delivery systems utilizing sol–gel nanocarrier encapsulation. These formulations, deployed as foliar sprays, slow-release dispensers, or smart volatile labels, are designed for controlled release and environmental stability, making them adaptable to a wide range of climatic and agronomic conditions, including arid regions where chemical control is limited or undesirable.

Future work will focus on: (1) multi-season field trials across diverse agroecological zones to assess performance consistency, crop compatibility, and ecological safety; (2) techno-economic evaluations to compare production costs, pest suppression efficacy, and yield outcomes relative to conventional pesticides; (3) decentralized and low-cost production models to support local implementation using biodegradable, scalable materials; and (4) participatory design with growers and stakeholders to enhance adoption and adapt formulations to specific crop-pest systems.

Together, these translational steps will bridge fundamental insights in chemical ecology with practical, on-farm applications. The proposed strategy offers a sustainable, scalable, and biologically informed alternative to synthetic insecticides, with broader relevance to other aphid-susceptible crops such as citrus, grapes, and Taif rose.

## Nomenclatures

*P. granatum*, *Punica granatum*; *A. punicae*, *Aphis punicae*; *C. undecimpunctata*, *Coccinella undecimpunctata*; *T. magnum*, *Tapinoma magnum*; VOCs, Volatile organic compounds; OLS, Open-loop stripping; GC–MS, Gas chromatography-mass spectrometry; RT, Retention time; S1-DVB, S2-DVB, S3-DVB, S4-DVB, Stage of field ecological systems 1, 2, 3, and 4, respectively; PCA, Principal component analysis; AIP, Aphid infested-pomegranate; G1: AIP, Pre-infested pomegranate after 24 h**;** G2: AIP-T, The tripartite community consists of pre-infested pomegranate after 48 h + *T. magnum* after 24 h.**;** G3: AIP-TC24, The quadripartite community consists of pre-infested pomegranate after 48 h + *T. magnum* after 24 h + *C. undecimpunctata* after 24 h.**;** G4: AIP-TC48, The quadripartite community consists of pre-infested pomegranate after 72 h + *T. magnum* after 48 h + *C. undecimpunctata* after 48 h.**;** °C, Degree Celsius; RH, Relative humidity ; h, Hour; µL, Microliter; mL, Milliliter; min, Minute; m/z, M stands for mass and Z stands for charge number of ions; |r|, Absolute value describes the distance from zero that a number is on the number line, without considering direction.; P, Wilcoxon Test; α, The threshold for statistical significance, often set at 0.05, representing a 5% risk of a Type I error.

## Data Availability

The sequencing data generated in this study were deposited in the National Center for Biotechnology Information (NCBI) repository: https://www.ncbi.nlm.nih.gov/nucleotide/ (accessed on 5 April 2022). The GenBank accession numbers are as follows: *Aphis punicae* - KSA-Taif MZ091379, *Tapinoma magnum* ON149799, *Coccinella undecimpunctata* ON149797.
