# Correction: Decoding chemical interactions among pomegranate, *Aphis punicae*, and associated insects in Taif fields through open-loop stripping

**DOI:** 10.3389/fpls.2025.1659820

**Published:** 2025-07-29

**Authors:** Frontiers Production Office

**Affiliations:** Frontiers Media SA, Lausanne, Switzerland

**Keywords:** *Punica granatum L.*, non-targeted analysis, Taif governorate, multivariate statistical analysis, GC-MS headspace, open-loop stripping, integrated pest management

There was a mistake in Figure 8 as published. Figure 4 was duplicated in Figure 8, the image has now been updated.

The original version of this article has been updated.

