# Supplementary material for: Decoding chemical interactions among pomegranate, Aphis punicae, and associated insects in Taif fields through open-loop stripping
Source: Front Plant Sci. 2025 Jun 26;16:1541538. doi: 10.3389/fpls.2025.1541538 (PMC12243111; doi:10.3389/fpls.2025.1541538)
Supplement: Supplementary file 1 [file DataSheet1.pdf]

Article

Supplementary Information

Decoding chemical interactions among pomegranate, *Aphis punicae*, and associated insects in Taif fields through open-loop stripping

Nour Houda M'sakni[1*†], Taghreed Alsufyani[1*†], Noura J. Alotaibi[2†]
[1]Department of Chemistry, College of Science, Taif University, P.O. Box 11099, Taif 21944, Saudi Arabia;
[2]Department of Biology, College of Science, Taif University, P.O. Box 11099, Taif 21944, Saudi Arabia;
* **Correspondence:** Nour Houda M'sakni (nour.h@tu.edu.sa); Taghreed Alsufyani (taghreed.alsufyani@tu.edu.sa).

Keywords: integrated pest management, *Punica granatum L.*, *Aphis punicae*, Taif Governorate, multivariate statistical analysis, GC-MS headspace, open-loop stripping

Sequencing Data and Accession Numbers:

The sequencing data generated in this study were deposited in the National Center for Biotechnology Information (NCBI) repository: **https://www.ncbi.nlm.nih.gov/nucleotide/** (accessed on 5 April 2022). The GenBank accession numbers are as follows:

Aphis punicae - KSA-Taif	MZ091379
Tapinoma magnum	ON149799
Coccinella undecimpunctata	ON149797

Supplementary Tables

Supplementary Table 1 Statistical Analysis of Volatile Organic Compounds (VOCs) Using Two-Factor ANOVA Without Replication – Traceability via Microsoft Excel

SUMMARY	*Count*	*Sum*	*Average*	*Variance*
bio1:o-Xylene	12	39.0349504	3.25291254	4.6
bio2:p-Xylene	12	89.5343114	7.46119262	10.9
bio3:1-Ethyl-3	12	7.56871993	0.63072666	0.5
bio4:1,3,5-Trir	12	21.8467444	1.82056203	4.5
bio5:1-Methy	12	46.0736954	3.83947462	49.9
bio6:m-Ethylc	12	32.3449025	2.69540854	28.1
bio7:2-Methy	12	51.037194	4.2530995	54.7
bio8:4-Heptar	12	246.022647	20.5018873	1124.5
bio9:Methyl n	12	2.33716094	0.19476341	0.1
bio10:Methyl	12	2.2879033	0.19065861	0.1
bio11:3-Carer	12	9.2099545	0.76749621	2.5
bio12:Sabiner	12	8.92302691	0.74358558	1.9
bio13:Tricycle	12	184.119391	15.3432826	776.0
bio14:Limone	12	93.3128717	7.77607264	204.4
bio15:(+)-4-Ca	12	6.52362575	0.54363548	1.0
bio16:Benzalc	12	96.4463223	8.03719352	68.2
bio17:alpha-N	12	0.13658782	0.01138232	0.0
bio18:2,2,4-tr	12	10.5774399	0.88145332	2.5
bio19:Unkno	12	57.5869258	4.79891049	89.4
bio20:Unknov	12	126.308135	10.5256779	196.5
bio21:Trideca	12	43.18736	3.59894667	19.2
bio22:Unknov	12	0.05556537	0.00463045	0.0
bio23:Unknov	12	0.05839495	0.00486625	0.0
bio24:Pentade	12	9.37772441	0.78147703	2.1
bio25:Heptad	12	9.27664355	0.77305363	2.0
bio26:1-Meth	12	6.77894077	0.56491173	1.1
bio27:Caryopl	12	37.6242107	3.13535089	35.4
bio28:beta-Fa	12	0.17722557	0.0147688	0.0
bio29:β-helmi	12	0.06173415	0.00514451	0.0
bio30:Unknov	12	0.03374468	0.00281206	0.0
G1: AIP	30	100	3.33333333	32.57601866
G1: AIP	30	100	3.33333333	34.68557267
G1: AIP	30	100	3.33333333	42.34489039
G2: AIP-T	30	100	3.33333333	236.1295855
G2: AIP-T	30	100	3.33333333	174.2102644
G2: AIP-T	30	100	3.33333333	196.9250468
G3: AIP-TC24	30	100	3.33333333	119.7243064
G3: AIP-TC24	30	100	3.33333333	154.2530771
G3: AIP-TC24	30	100	3.33333333	128.5195425
G4: AIP-TC48	30	137.864055	4.59546849	89.40904009
G4: AIP-TC48	30	100	3.33333333	47.41659206
G4: AIP-TC48	30	100	3.33333333	47.22820199

ANOVA

urce of Variati	*SS*	*df*	*MS*	*F*	*P-value*	*F crit*
Rows	8359.01215	29	288.241798	3.123247818	4.1731E-07	1.5031683
Columns	43.807092	11	3.98246291	0.04315203	0.99999888	1.81872756
Error	29440.2299	319	92.2891218			
Total	37843.0491	359				

Supplementary Table 2 ANOVA Summary of biomarkers categorized by Frequency range (F), excluding group membership information; Analysis indicating reduction of biomarkers by Frequency value from 30 to 24, divided into four categories without specifying corresponding biological interaction groups. PCA revealed that 20 biomarkers were responsible for the separation of biological interaction groups, as shown in Supplementary Table 3.

	SUMMARY	*Count*	*Sum*	*Average*	*Variance*
Biomarkers 1 (F > 200)	bio8:4-Heptanone	12	246.022647	20.5018873	1124.5
	bio13:Tricyclene	12	184.119391	15.3432826	776.0
Biomarkers 2 (100<F < 200)	bio14:Limonene	12	93.3128717	7.77607264	204.4
	bio20:Unknown alkane-3	12	126.308135	10.5256779	196.5
Biomarkers 3 (50 <F < 100)	bio19:Unknown alkane-2	12	57.5869258	4.79891049	89.4
	bio16:Benzaldehyde	12	96.4463223	8.03719352	68.2
	bio7:2-Methylnaphtalene	12	51.037194	4.2530995	54.7
	bio5:1-Methyl-3-tert-butylbenzene	12	46.0736954	3.83947462	49.9
Biomarkers 4 (10 <F < 50)	bio27:Caryophyllene	12	37.6242107	3.13535089	35.4
	bio6:m-Ethylcumene	12	32.3449025	2.69540854	28.1
	bio21:Tridecane	12	43.18736	3.59894667	19.2
	bio2:p-Xylene	12	89.5343114	7.46119262	10.9
Biomarkers 5 (F < 5)	bio1:o-Xylene	12	39.0349504	3.25291254	4.6
	bio4:1,3,5-Trimethylbenzene	12	21.8467444	1.82056203	4.5
	bio18:2,2,4-trimethyl-Pentane	12	10.5774399	0.88145332	2.5
	bio11:3-Carene	12	9.2099545	0.76749621	2.5
	bio24:Pentadecane	12	9.37772441	0.78147703	2.1
	bio25:Heptadecane	12	9.27664355	0.77305363	2.0
	bio12:Sabinene	12	8.92302691	0.74358558	1.9
	bio26:1-Methyl-1H-imidazole	12	6.77894077	0.56491173	1.1
	bio15:(+)-4-Carene	12	6.52362575	0.54363548	1.0
	bio3:1-Ethyl-3-methylbenzene	12	7.56871993	0.63072666	0.5
	bio9:Methyl methanoate	12	2.33716094	0.19476341	0.1
	bio10:Methyl Salicylate	12	2.2879033	0.19065861	0.1

Supplementary Table 3 Essential and semiessential biomarkers of pattern quadruplet interaction between Aphid infested plant-Ant-Ladybird. The strategy used to identify essential and semiessential biomarkers was explained in our previous study (Alotaibi et al., 2023).

		Triangle: essential biomarkers	Circle: semiessential biomarkers	Intermediar biomarkers
G-1	*PIN24-DVB*	**Bio5:1-Methyl-3-tert-butylbenzene<br>Bio6:m-Ethylcumene<br>Bio11:3-Carene<br>Bio15:(+)-4-Carene<br>Bio18:2,2,4-trimethyl-Pentane<br>Bio22:Unknown alkane-3<br>Bio27:Caryophyllene**	**Bio17:alpha-Methylbenzeneacetaldehyde<br>Bio23:Unknown alkane-4<br>Bio28:beta-Farnesene**	**Bio7:2-Methylnaphtalene<br>Bio16:Benzaldehyde**
G-2	*PIN48An24-DVB*	**Bio9:Methyl methanoate<br>Bio19:Unknown alkane-1**	-	-
G-3	*PIN48An24Lb24-DVB*	**Bio8:2-Heptanone**	**Bio13:Tricyclene**	-
G-4	*PIN728An48Lb48-DVB*	**Bio3:1-Ethyl-3-methylbenzene<br>Bio4:1,3,5-Trimethylbenzene<br>Bio10:Methyl Salicylate**	**Bio29:β-helmiscapene (β-Selinene)**	**Bio1:o-Xylene<br>Bio2:p-Xylene<br>Bio21:Tridecane**

		**Bio12:Sabinene**<br>**Bio14:Limonene**<br>**Bio20:Unknown alkane-2**<br>**Bio24:Pentadecane**<br>**Bio25:Heptadecane**<br>**Bio26:1-Methyl-1H-imidazole**<br>**Bio30:Unknown-1**		

Supplementary Figures

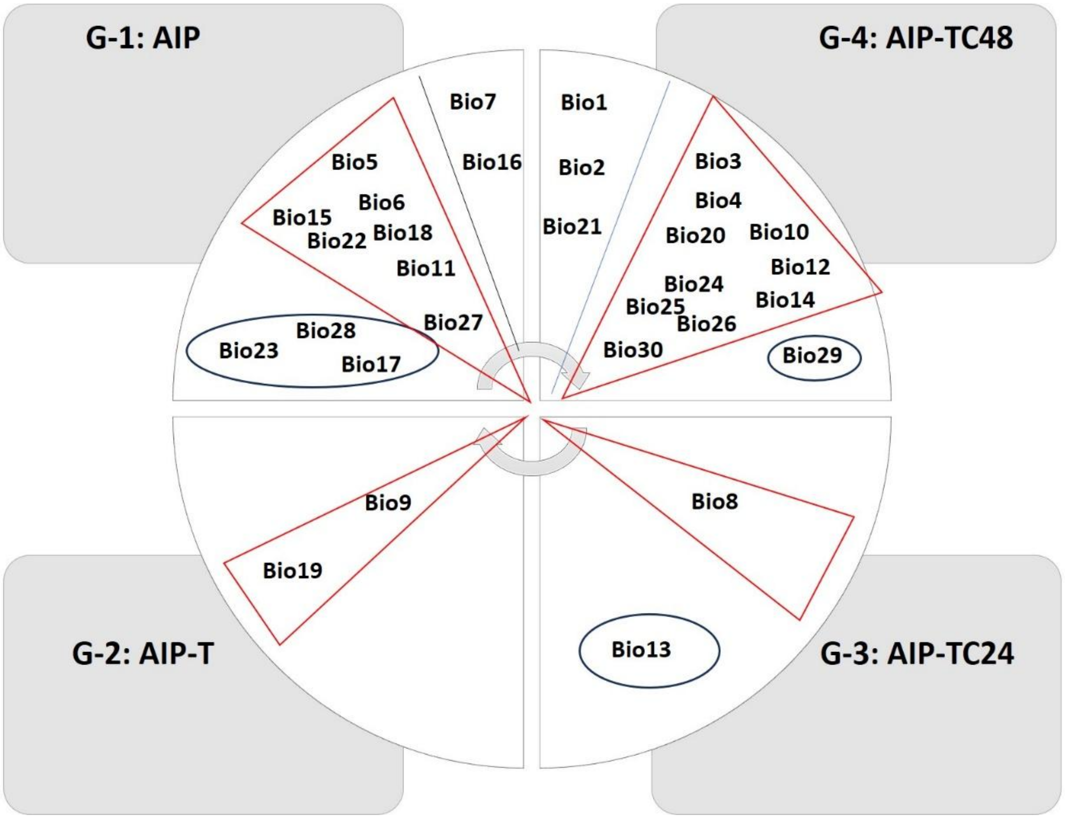

Supplementary Figure 1. Essential (triangle) and semiessential (circle:) biomarkers of pattern quadruplet interaction between Aphid infested plant-Ant-Ladybird. The strategy used to identify essential and semiessential biomarkers was explained in our previous study (Alotaibi et al., 2023).

Supplementary References

Alotaibi, N. J., Alsufyani, T., M'sakni, N. H., Almalki, M. A., Alghamdi, E. M., & Spiteller, D. (2023). Rapid Identification of Aphid Species by Headspace GC-MS and Discriminant Analysis. *Insects, 14*(7), 589. Retrieved from https://www.mdpi.com/2075-4450/14/7/589
